# A biophysical framework for conscious state transitions based on intrinsic neuronal properties: the dynamic contextual element memory (DCEM) model

**DOI:** 10.3389/fnsys.2026.1894336

**Published:** 2026-09-28

**Authors:** Takayoshi Tsubo

**Affiliations:** Brain Basic Function Laboratory, Hachioji, Japan

**Keywords:** beta oscillation, contextual element, dendritic spine, gamma oscillation, neuromodulation, working memory

## Abstract

Unlike artificial systems that execute externally specified commands, the brain constructs contextual representations to guide behaviors that satisfy innate and learned desires generated by the Affective Valuation System (AVS). However, the neural mechanisms by which contextual representations are formed, selected, maintained, and replaced to generate conscious state transitions remain unknown. Here, we propose the Dynamic Contextual Element Memory (DCEM) model as a biophysical framework within the dorsolateral prefrontal cortex. The DCEM is conceived as a dynamic contextual memory consisting of numerous logical Contextual Elements (CEs), each implemented by multiple physical cortical columns. AVS signals initiate contextual exploration in the DCEM and guide contextual selection, maintenance, and transition. Recurrent interactions between the DCEM and the parietal and temporal association cortices generate γ-band contextual candidates from sensory and memory information. Physical columns of candidates selected by Contextual Element Decision Neurons (CEDeN) in the mediodorsal thalamus receive synchronized feedback and form a coherent β-band Contextual Element Loop (CE Loop) representing a single logical Contextual Element. Outputs from DCEM Layer 5 redistribute the selected context throughout the association cortices, allowing AVS to shape candidates. CE Loops that lose CEDeN support following re-evaluation of behavioral outcomes or new information fail to maintain synchrony and disappear. We therefore propose that conscious state transitions occur through the formation, maintenance, disappearance, and replacement of CE Loops. This model was naturally derived by extending the intrinsic biophysical properties of neurons identified in our studies to cortical networks while integrating accumulated findings from modern observational techniques, neuroanatomy, neurophysiology, and neuroscience.

## 1 Introduction

Unlike artificial systems that passively execute externally given commands, the brain, driven by continuous desires based on biological needs and experience-dependent values, explores, selects, and integrates sensory information, memory information, and internal information to construct contexts that guide judgment, language, and behavior (Aston-Jones and Cohen, 2005; Menon and Uddin, 2010; Miller and Cohen, 2001; Padoa-Schioppa, 2011; Schultz, 2016). However, how this context construction and conscious-state transition are realized by neurons, cortical columns, and cortico-thalamic circuits remains insufficiently understood (Dehaene and Changeux, 2011; Halassa and Kastner, 2017; Halassa and Sherman, 2019; Mashour et al., 2020; Wolff and Halassa, 2024).

EEG and MEG studies have demonstrated γ-band and β-band activities associated with consciousness, attention, working memory, and decision-making, while fMRI studies have revealed widespread activity in the prefrontal cortex, parietal and temporal association cortices, thalamus, and subcortical structures (Fries, 2005; Halassa and Kastner, 2017; Logothetis, 2008; Lundqvist et al., 2016; Lundqvist et al., 2018; Mashour et al., 2020; Spitzer and Haegens, 2017; Wolff and Halassa, 2024). However, because EEG and MEG cannot sufficiently localize activity at the level of cortical layers and cortical columns, and fMRI cannot track exploration, selection, and state transitions on the millisecond timescale, the neural mechanisms leading from desire to context construction remain unclear (Logothetis, 2008).

Global Neuronal Workspace Theory, Predictive Processing, Recurrent Processing Theory, and attractor network models explain important aspects of consciousness and cognition (Dehaene and Changeux, 2011; Friston, 2010; Hopfield, 1982; Mashour et al., 2020; Wang, 2001). However, they do not sufficiently explain the neural mechanisms by which internally generated desires initiate large-scale information exploration, select candidate representations, and organize them into temporally continuous contexts (Dehaene and Changeux, 2011; Friston, 2010; Mashour et al., 2020).

In the present study, we propose the Dynamic Contextual Element Memory (DCEM) model centered on the dorsolateral prefrontal cortex (DLPFC), as shown in Figure 1, as a biophysical framework for addressing this problem. DCEM is defined not as a static memory device but as a dynamic cortico-thalamic neural system that explores and integrates distributed information and forms, maintains, and updates context according to desires.

**FIGURE 1:**
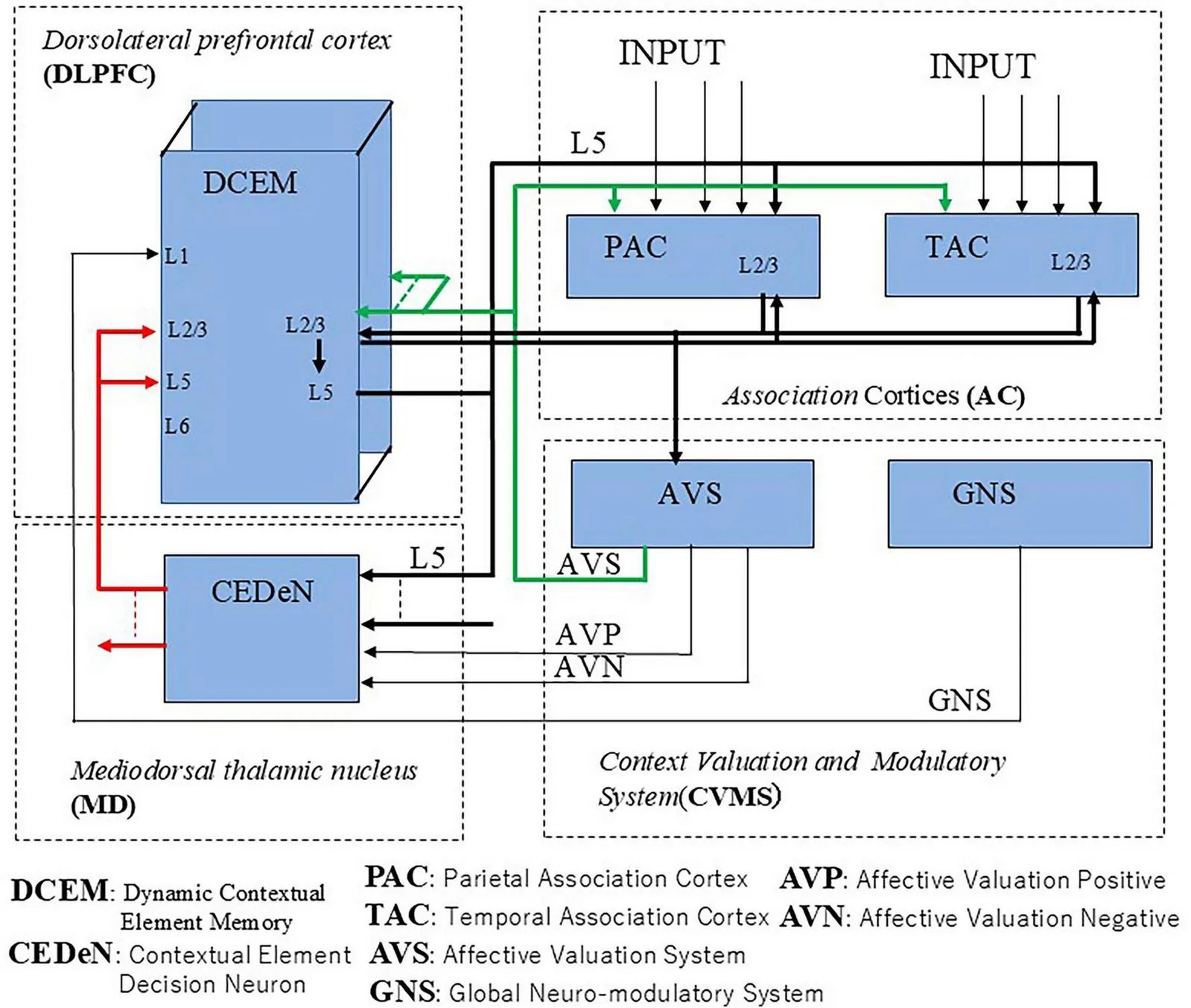
Dynamic Contextual Element Memory (DCEM) model and pathways of the γ-band recurrent loop and β-band CE Loop. Outputs from the Affective Valuation System (AVS) project to the Dynamic Contextual Element Memory (DCEM) and to Layer 2/3 (L2/3) of the parietal and temporal association cortices (PAC/TAC). Outputs from L2/3 of PAC/TAC provide inputs to L2/3 of DCEM. Layer 5 (L5), which receives outputs from Layer 3 (L3), projects its outputs to L2/3 of PAC/TAC and to the CEDeN. These connections form a γ-band recurrent loop. In contrast, when the AVS, which monitors the output of PAC/TAC L2/3, sends a positively evaluated AVP signal to the CEDeN, feedback from the CEDeN, indicated by the thick red lines, is returned simultaneously to L2/3 of multiple physical columns constituting the logical column of the corresponding CE. This causes the delayed L3 outputs to be sent to L5. Consequently, the delayed L5 outputs from the multiple physical columns are projected simultaneously, thereby forming a synchronized β-band CE Loop. The mechanism by which L3 output is delayed when DCEM L2/3 simultaneously receives input from the CEDeN is described in section “3.5 Local microcircuit dynamics underlying β-band periodicity.”

In the DCEM model, the context that guides behavior to fulfill internal desires is considered to potentially include functionally distinct types of information concerning who or what, does what, to what, in what state, when, where, why, and how. Accordingly, in the present study, Subject, Verb, Object, Complement, When, Where, Why, and How (Attribute) are assumed as representative Contextual Element Categories (CE Categories) to organize the functional roles of such contextual information.

These do not correspond directly to natural-language grammatical categories in the nervous system, but are model-based classifications representing the functional roles of information constituting a context. Each category consists of multiple Contextual Elements (CEs), and the information corresponding to the specific contents of these CEs is assumed to exist in a distributed manner as numerous Meaning Groups formed through experience within the Association Cortices (AC).

As a specific structural example of the present model, each CE Category is assumed to consist of approximately 25 CEs, with approximately 200 logical CEs handled throughout DCEM. Each CE is assumed to correspond to one logical column, and each logical column is further assumed to consist of approximately 10 physical columns (Mountcastle, 1997; Petersen, 2007). Therefore, the values of approximately 200 logical CEs and approximately 2,000 physical columns are not currently established anatomical constants, but model assumptions introduced to concretely describe the structure and operation of DCEM.

The DCEM model is positioned as a cortico-thalamic neural mechanism that forms the context necessary to satisfy desires continuously provided by the Affective Valuation System (AVS) and links that context to appropriate behavior. The AVS includes an innate Proto-AVS, present from birth and related to survival functions such as hunger, thirst, thermoregulation, and fear, as well as a higher-order AVS formed and developed through postnatal experience and learning. In the present model, these value states are considered to continuously drive context formation by DCEM.

To satisfy AVS desires, the process of selecting currently necessary information from the large amount of information present in the perceptual and temporal association cortices (PAC/TAC) and constructing an executable context can broadly be divided into two stages of recurrent loops. In both cases, as indicated by Basic Property 2 in section “1.1 Fundamental properties of neurons,” recurrent loop structures predominate so that output does not cease.

**First Stage: Formation of Asynchronous γ-Band Candidate Loops**

**Stage 1: Inputs from PAC/TAC, AVS, and CEDeN to DCEM**

As shown in Figure 1, Layer 2/3 (L2/3) of DCEM receives primarily outputs from L2/3 of PAC/TAC, which has diverse input pathways, outputs from AVS, and outputs from the Contextual Element Decision Neuron (CEDeN), described below.

**Stage 2: Widespread Outputs from DCEM Layer 5**

Numerous large Layer5 (L5) pyramidal neurons receiving inputs from Layer 3 (L3) of DCEM project to widespread regions, including L2/3 of PAC/TAC and CEDeNs, which are hypothesized to exist within the thalamic MD nucleus.

**Stage 3: Formation of Recurrent Loops**

Through these connections, as shown in Figure 1, the following recurrent loop is considered to be formed:

**PAC/TAC L2/3 → DCEM L2/3 → DCEM L5 → PAC/TAC L2/3**

Meanwhile, outputs from DCEM L5 are also sent in parallel to CEDeNs. Which recurrent loops are formed depends on changes in the input information to DCEM L2/3 and the input information to PAC/TAC L2/3.

**Stage 4: Replacement of Recurrent Loops in Response to Inputs**

When new recurrent loops are formed in response to changes in input, some of the recurrent loops that had previously been active in their vicinity are considered to cease, resulting in replacement of recurrent loops according to the input content. This mechanism is considered to involve inhibitory interactions between L2/3 neurons of each physical column and L2/3 neurons of neighboring columns (Helmstaedter et al., 2009). In contrast, when there is no substantial change in the input states to L2/3 of DCEM and PAC/TAC, the same recurrent loops may continue to remain active.

**Stage 5: Parallel Formation of Numerous Recurrent Loops**

In the present study, one logical CE is assumed to consist of approximately 10 physical columns, and approximately 200 logical CEs are assumed to exist throughout DCEM. Therefore, in this structural example, approximately 2,000 physical columns exist, and numerous recurrent loops involving these columns can potentially be formed. However, considering inhibitory interactions between neighboring columns, not all of these are considered to be active simultaneously; rather, a subset is selected and continues to oscillate in parallel.

**Stage 6: Diverse Combinations of Inputs**

L2 and L3 pyramidal neurons receive numerous synaptic inputs. As shown in section “3.2 Charge-retention properties of dendritic spines and extended synaptic integration,” item 4, by utilizing these numerous input sites, DCEM L2/3 neurons can receive diverse information sent from PAC/TAC in various combinations (Tsubo, 2021). This diversity of combinations is considered to enable the formation of numerous different contextual candidates.

**Stage 7: Asynchronous γ-Band Candidate Loops**

As described above, the state in which numerous recurrent loops receiving inputs from PAC/TAC to DCEM and returning from DCEM L5 to PAC/TAC continue to oscillate independently without being synchronized with one another is positioned in the present study as γ-band candidate loops.

**Stage 8: Candidate Sets of CE Contents**

Thus, the state in which numerous γ-band candidate loops are formed, disappear, and are replaced in parallel is considered to correspond to a state in which numerous specific contents that could enter each CE are being explored as candidates. In the following second stage, among these candidates, CEs evaluated by the AVS are selected by CEDeNs, and β-band CE Loops are formed through synchronization of the corresponding groups of physical columns.

**Second Stage: Formation of Synchronized β-Band CE Loops**

Because information within the cerebral cortex is handled in coded form, correct decoding of that information requires either maintaining the same information for a prolonged period or ensuring temporal consistency, namely synchronization, among the multiple data elements constituting the code, as indicated by Basic Property 4 in section “1.1 Fundamental properties of neurons.” In the present model, the β-band CE Loop is positioned as the mechanism that forms this synchronized information state.

**Stage 1: Monitoring of Candidate Information by the AVS**

The AVS is considered to project outputs to DCEM while simultaneously monitoring, as shown in Figure 1, the γ-band coded candidate information continuously transmitted from PAC/TAC to DCEM.

**Stage 2: Output of AVS Evaluation Results to CEDeNs**

As shown in Figure 1, for the coded candidate information transmitted from PAC/TAC to DCEM, Affective Valuation Positive (AVP) signals are considered to be sent to CEDeNs according to items consistent with the desires and value states of the AVS, whereas Affective Valuation Negative (AVN) signals are considered to be sent to CEDeNs according to items that conflict with the desires or value states of the AVS.

**Stage 3: Input Integration in CEDeNs**

Each CEDeN receives inputs from L5 of multiple physical columns constituting the corresponding logical CE, together with multiple AVP and AVN signals from the AVS. CEDeNs integrate these inputs. As described in detail regarding CEDeN input integration in sections “1.6 Initiation and termination mechanisms of contextual element loops” and “3.4 Threshold model of the contextual element decision neuron (CEDeN),” because the present model assumes that a CEDeN corresponding to each logical CE exists, in the example configuration of approximately 200 logical CEs used in the present study, approximately 200 CEDeNs are assumed to independently perform input integration.

**Stage 4: Initiation of Synchronized Output from CEDeNs**

In the γ-band candidate-loop state, L5 inputs sent to a CEDeN from multiple physical columns constituting the same logical CE are asynchronous with one another; therefore, summation of these inputs alone is considered unlikely to raise the integrated value of the CEDeN to the functional threshold. However, when the integrated value exceeds the threshold through the addition of sufficient AVP signals from the AVS, the CEDeN sends synchronized outputs to L2/3 of the multiple physical columns constituting the corresponding logical CE, as indicated by the thick red lines in Figure 1.

**Stage 5: Initiation of Synchronized β-Band CE Loops**

In multiple physical columns receiving simultaneous inputs from a CEDeN, L3 output is delayed by inhibitory effects mediated through L2, and L5 output is consequently also delayed. As a result, L5 outputs with a longer period than the γ-band are emitted nearly simultaneously from L5 of the corresponding group of multiple physical columns. In the present model, this is considered the initiation of a synchronized β-band CE Loop. Therefore, the basic pathway of the β-band CE Loop is a recurrent loop passing through the connections indicated by the red lines in Figure 1:

DCEM L2/3 → delayed L3 → DCEM L5 → PAC/TAC L2/3 and CEDeN

Thus, the selection and synchronization mechanism involving feedback to the CEDeN is maintained. The period of the β-band CE Loop is considered to be determined primarily by the delay generated in L3. The local circuit mechanism underlying the delay in L3 is described in Chapter 3. Meanwhile, because the present DCEM model assumes approximately 2,000 physical columns constituting the logical columns of approximately 200 CEs throughout the system, many γ-band CE candidate loops still remain between PAC/TAC and DCEM.

**Stage 6: Maintenance and Termination of β-Band CE Loops**

Once a β-band CE Loop is initiated, the L5 outputs of multiple physical columns constituting the same logical CE become synchronized. Consequently, these L5 inputs are temporally summed in the CEDeN, making it easier for the summed L5 inputs alone to maintain the integrated value of the CEDeN above the functional threshold. Therefore, once a β-band CE Loop has been initiated, its maintenance is considered to continue through synchronized L5 inputs without being strongly dependent on AVP signals from the AVS.

In contrast, when the content of PAC/TAC changes because of new sensory information, memory information, or behavioral outcomes, and the number of items conflicting with AVS evaluation increases, AVN signals increase. If this causes the integrated value of the CEDeN to fall below the functional threshold, synchronized output from the CEDeN ceases, and the corresponding β-band CE Loop also terminates.

Thus, in the present model, AVP plays an important role in the selection process that initiates the transition from a γ-band candidate loop to a β-band CE Loop, whereas once formed, a β-band CE Loop is maintained relatively stably by synchronized L5 inputs and is terminated by an increase in AVN.

Accordingly, the formation, maintenance, and termination of β-band CE Loops occur independently for each CE.

The simultaneous maintenance of β-band CE Loops corresponding to multiple CEs forms the contextual state at that time. In contrast, when sensory information, memory information, behavioral outcomes, or AVS evaluations change, β-band CE Loops corresponding to CEs that have lost support terminate and are replaced by β-band CE Loops of newly selected CEs. Therefore, even when a major goal or desire generated by the AVS persists, the specific CE contents required to achieve that goal can be sequentially replaced.

Taken together, the transition from the γ-band to the β-band in DCEM does not merely represent a change in the oscillation frequency of recurrent loops. Diverse contextual candidates are explored through numerous asynchronous γ-band candidate loops; among them, necessary CEs are selected through value evaluation by the AVS and input integration by CEDeNs, and the corresponding groups of physical columns are formed and maintained as synchronized β-band CE Loops.

Individual CE Loops then terminate and are replaced in response to changes in conditions. This continuous sequence of candidate formation, selection, synchronization, maintenance, termination, and replacement at the CE level is considered to realize dynamic context formation and conscious-state transitions in DCEM.

### 1.1 Fundamental properties of neurons

To understand complex brain functions as a system, a framework based not only on individual neuronal phenomena but also on fundamental principles that integrate them is required (Dayan and Abbott, 2001; Sejnowski et al., 1988). However, in previous studies, the relationship between the biophysical properties of single neurons and dynamic state transitions in large-scale neural networks has rarely been organized from a unified perspective, and consequently, the system-level understanding of neural activity remains limited (Sejnowski et al., 1988).

In the present study, the following six fundamental properties supporting the operation of neuronal networks are assumed as the basis of the discussion. These properties provide a conceptual foundation for an integrated understanding of individual phenomena, and their detailed physical and physiological bases are described in Chapter 3.

**Fundamental Property 1: High-Dimensional and Nonlinear Synaptic Integration**

Pyramidal neurons possess numerous dendritic spines and integrate large numbers of synaptic inputs in their dendrites and spines (London and Häusser, 2005; Stuart and Spruston, 2015; Yuste and Denk, 1995). Furthermore, based on our previous studies, we consider that the charges retained in spines and the electrical properties of the spine neck and intracellular fluid contribute to the integration of input signals arriving within a certain time window (Tsubo, 2021).

Interactions between excitatory and inhibitory inputs produce condition-dependent and selective response properties (Abbott and Regehr, 2004; Tsubo, 2024), and neurons are considered to possess computational functions capable of responding to enormous combinations of inputs as threshold-type nonlinear selectors in a high-dimensional input space, rather than functioning as fixed logical elements (Fusi et al., 2016; Rigotti et al., 2013; Tsubo, 2021, 2023, 2024).

**Fundamental Property 2: Transient Output and Mechanisms for Initiation and Termination of Recurrent Loops**

Neuronal output is fundamentally transient and is generated as an action potential only when threshold conditions are satisfied (Izhikevich, 2007; Lisman, 1997; Tsubo and Kurokawa, 2018; Tsubo, 2021). Recurrent circuit structures are important for maintaining persistent activity states within the cortex, and recurrent activity can generate persistent neural states (Amit, 1995; Wang, 2001). In the present model, for example, γ-band recurrent loops are considered to form between DCEM and PAC/TAC. However, because L2/3 has inhibitory connections with neighboring columns, the initiation of a new recurrent loop is considered to terminate previously active recurrent loops in neighboring columns through repeated inhibitory effects (Helmstaedter et al., 2009). Thus, γ-band recurrent loops are assumed to be readily switched to new loops in response to new inputs.

**Fundamental Property 3: Conditions for Action Potential Generation**

Two types of conditions for action potential generation are considered: generation through temporal and spatial integration of excitatory postsynaptic potentials (EPSPs) occurring in close temporal proximity, and generation through an increase in membrane potential determined by the total amount of charge retained within the neuron and the total membrane capacitance (Tsubo and Kurokawa, 2018; Tsubo, 2021).

Accordingly, the total amount of charge retained within spines distributed throughout the dendritic arbor extending from Layer 1 to Layer 5 is considered to contribute to action potential generation (Tsubo, 2021). Repeated projections caused by loop oscillations are also considered to increase the amount of charge accumulated within spines and to activate neurons that had previously been in an inactive state (Tsubo, 2021).

**Fundamental Property 4: Distributed Encoding and Phase Alignment**

Information in the cerebral cortex is distributedly encoded, and meaning is constructed through combinations of activity patterns across multiple neuronal populations (Fusi et al., 2016; Rigotti et al., 2013; Tsubo, 2023, 2024). Such mixed selectivity enables an extremely large state space to be realized even with a finite number of neurons; however, for this information to function as a unified state, temporal alignment among widely distributed neuronal populations is important (Engel et al., 2001; Fries, 2005; Singer, 1999; Varela et al., 2001; Tsubo, 2023, 2024).

In the present model, synchronized β-band CE Loops formed by CEDeNs are assumed to provide temporal consistency to coded contextual information distributed across multiple physical columns, thereby allowing downstream external output systems to decode and use that information as a coherent state. Through this synchronization, contextual information distributed across multiple physical columns is considered to be output while maintaining temporal consistency and to be converted into specific behavior.

**Fundamental Property 5: Rapid and Low-Attenuation Action Potential Conduction**

For neuronal networks to perform rapid information processing, action potentials must propagate rapidly and with low attenuation along axons. Axon diameter, membrane properties, and the myelin sheath play important roles in action potential conduction (Ritchie and Rogart, 1977; Tsubo and Kurokawa, 2018). In our previous study, we proposed a new axonal equivalent circuit that incorporates a capacitive component in the axial direction by considering the high dielectric properties of intracellular fluid, and analyzed its relationship with rapid and low-attenuation signal conduction (Tsubo and Kurokawa, 2018).

**Fundamental Property 6: Evolutionarily Determined Macroarchitecture**

The cerebral cortex is constructed on the basis of a fundamental macroarchitecture formed through evolutionary and developmental processes (Douglas and Martin, 2004; Harris and Shepherd, 2015; Mountcastle, 1997; Rakic, 1988). For example, the basic functional differentiation of sensory areas such as the visual, auditory, and somatosensory cortices, as well as their broad connection patterns with the primary motor cortex, are formed during early development (Harris and Shepherd, 2015; Markram et al., 2015; Mountcastle, 1997; Rakic, 1988). In contrast, within this framework, synaptic connection strengths, circuit refinement, and functional differentiation develop through postnatal experience and learning (Fusi et al., 2016; Kasai et al., 2010; Markram et al., 2015; Yuste and Denk, 1995). For example, in language function, the capacity for language acquisition and the basic organization of language networks are influenced by innate factors, whereas the particular language acquired, its vocabulary, grammar, and semantic system are formed by the postnatal environment (Fedorenko and Blank, 2020; Hickok and Poeppel, 2003). Thus, learning and neural plasticity are considered to functionally refine the internal structure of an innate macroarchitecture (Fusi et al., 2016; Kasai et al., 2010; Markram et al., 2015; Yuste and Denk, 1995).

### 1.2 Context valuation and modulatory system (CVMS)

In conscious-state transitions, selection of contextual content and modulation of its activity state are important (Miller and Cohen, 2001; Rolls, 2016). In the present study, we introduce the Context Valuation and Modulatory System (CVMS) as an integrated framework responsible for these functions. CVMS is assumed to consist of the Affective Valuation System (AVS), which drives context formation and evaluates the value of contextual content, and the Global Neuromodulatory System (GNS), which modulates the activity state of the entire network.

#### 1.2.1 Promotion of context formation and evaluation of contextual elements by the AVS

**(1) Promotion of Context Formation by the AVS**

The Affective Valuation System (AVS) is considered to consist of two types of valuation systems with different origins and developmental processes.

The first is the innate Proto-AVS, present from birth, which is based on neural systems related to survival, danger avoidance, rewards, and motivation, including the hypothalamus, amygdala, brainstem, periaqueductal gray, and ventral striatum, and is considered to generate fundamental desires directly related to self-preservation and preservation of the species (Menon and Uddin, 2010; Padoa-Schioppa, 2011; Schultz, 2016).

The second is the experience-dependent AVS, which develops through postnatal experience and learning and is considered to form higher-order values and desires, such as experience-based values, social values, future goals, and intellectual interests, on the basis of a broad network including the orbitofrontal cortex, ventromedial prefrontal cortex, anterior cingulate cortex, insular cortex, and hippocampus (Eichenbaum, 2017; Menon and Uddin, 2010; Padoa-Schioppa, 2011; Rolls, 2016).

In the present model, the outputs of these two types of AVS are assumed to be mutually integrated and projected to DCEM and PAC/TAC, as shown in Figure 1, as continuous desire signals indicating the direction that should currently be prioritized. These signals are considered not only to serve as the driving force that initiates and maintains contextual exploration and generation in DCEM, but also to selectively activate sensory information and memory representations related to those desires in PAC/TAC.

**(2) Evaluation of Contextual Elements by the AVS**

As shown in Figure 1, the AVS is assumed to monitor the large amount of information transmitted from PAC/TAC to DCEM and continuously compare it with AVS desires. When the information is consistent with a desire, the positive evaluation result is sent to the CEDeN as Affective Valuation Positive (AVP), whereas when it conflicts with the desire or value state, the negative evaluation result is sent to the CEDeN as Affective Valuation Negative (AVN).

When AVP output is strong, formation of the CE Loop corresponding to that CE is promoted, whereas when AVN output is strong, the corresponding CE Loop is considered to terminate. For example, for a CE related to food, AVP may become predominant for food representations appearing in PAC/TAC during hunger, whereas AVN may increase for the same representation when the individual is satiated.

AVS monitors numerous γ-band recurrent loops that arise asynchronously and independently; however, because the contents of recurrent loops do not change when there is no change in the inputs to PAC/TAC and DCEM, relatively stable evaluation is considered possible (Tsubo, 2023, 2024).

#### 1.2.2 Modulation of global operating conditions by the GNS

The Global Neuromodulatory System (GNS) is a concept that integrates broad neuromodulatory systems as a functional system that modulates the activity state of the entire cortical network in the present model (Aston-Jones and Cohen, 2005; Avery and Krichmar, 2017; McCormick and Bal, 1997). The GNS modulates the operating conditions of each cortical column, namely sensitivity to synaptic input (gain), synchronization of neural activity (synchrony), and input responsiveness, thereby influencing the information-processing state of the entire cortex (Aston-Jones and Cohen, 2005; Avery and Krichmar, 2017).

This modulation involves broad neuromodulatory systems such as the noradrenergic system (locus coeruleus), dopaminergic system (ventral tegmental area), serotonergic system (raphe nuclei), and cholinergic system (basal forebrain) (Aston-Jones and Cohen, 2005; Avery and Krichmar, 2017; McCormick and Bal, 1997). The noradrenergic system is involved in modulation of arousal level and attentional gain (Aston-Jones and Cohen, 2005), whereas the dopaminergic system is involved in rewards- and value-related signals (Padoa-Schioppa, 2011; Schultz, 2016). The serotonergic system is also involved in broad modulation of neural activity states (Avery and Krichmar, 2017), and the cholinergic system is involved in modulation of cortical activity and sensory responsiveness (Avery and Krichmar, 2017; McCormick and Bal, 1997).

These neuromodulatory inputs affect dendritic integration properties and excitability of cortical pyramidal neurons (London and Häusser, 2005; Major et al., 2013; Stuart and Spruston, 2015). Based on Fundamental Property 3 proposed in the present study, GNS inputs to distal dendrites, including those in Layer 1, are also considered to contribute to the membrane potential and firing conditions of the corresponding pyramidal neurons (Tsubo, 2021).

An important point in the present study is that the GNS is not a mechanism that determines contextual content itself. Whereas the AVS primarily determines the direction of value evaluation of contextual candidates and the selection, maintenance, and termination of CE Loops, the GNS is considered to modulate the neural activity conditions that facilitate establishment of the selected state. In other words, whereas the AVS determines “what to select,” the GNS modulates “how strongly and how synchronously the state can be initiated and maintained.” The effects of the GNS become apparent during concentrated states such as escape, defense, attack, and sports.

The functional state of the GNS is not fixed and may change through experience and long-term neuromodulatory states (Aston-Jones and Cohen, 2005; Avery and Krichmar, 2017). Therefore, changes in neuromodulatory systems associated with arousal, stress states, motivation, and related factors may affect the conditions under which candidate formation and CE Loop formation occur in DCEM.

This division of roles between the AVS and GNS is important in the mechanism proposed in the present study for the transition from γ-band activity to β-band activity. In the γ-band, multiple contextual candidates are formed in parallel and are evaluated by the AVS, whereas the GNS is considered to modulate the gain and synchronization conditions of the entire circuit. Under these modulatory conditions, CEDeN-mediated selection and synchronized feedback are assumed to promote the transition of the selected activity state to β-band synchronization, thereby forming and maintaining a stable CE Loop.

Broad modulation by the GNS affects not only the cerebral cortex but also memory-related circuits including the hippocampal dentate gyrus (DG; Avery and Krichmar, 2017; Hainmueller and Bartos, 2020). In the present study, we consider the possibility that this modulatory mechanism operates in coordination with the formation, maintenance, and termination of CE Loops in DCEM and that changes in the overall circuit state produced by the GNS may also alter the conditions for initiating episodic-memory encoding in the DG (Eichenbaum, 2017; Hainmueller and Bartos, 2020; Yang et al., 2024).

Thus, conscious-state transitions and memory formation may be linked through a common functional system consisting of value evaluation of contextual candidates by the AVS and modulation of global operating conditions by the GNS.

### 1.3 Association-cortical column structure supporting DCEM dynamics

As described above, coded Meaning Groups are considered to be generated in parallel as γ-band activity in the temporal association cortex (TAC) and Perceptual Association Cortex (PAC). In the present model, these distributed candidate representations are assumed to undergo value evaluation and neuromodulation by the Context Valuation and Modulatory System (CVMS), after which CEDeN-mediated selection and synchronized feedback promote the transition of selected activity to a synchronized β-band state, thereby forming a stable contextual state.

However, establishment of such a dynamic contextual state requires a physical structural unit that integrates and synchronizes distributed neural activity and recursively maintains that state. In the present study, we focus on the cortical column structure of association cortices, including the dorsolateral prefrontal cortex (DLPFC), as this fundamental structural unit (Douglas and Martin, 2004; Harris and Shepherd, 2015; Markram et al., 2015; Mountcastle, 1997).

The cerebral cortex is a highly organized neural circuit consisting of layered and columnar structures (Douglas and Martin, 2004; Harris and Shepherd, 2015; Markram et al., 2015). In the DCEM model, functional interactions among Layer 1 (L1), Layer 2/3 (L2/3), Layer 5 (L5), and Layer 6 (L6) are considered to constitute the principal basis supporting dynamic contextual-state transitions.

**(1) L1: Broad Modulatory Input Layer from the CVMS**

Inputs from broad neuromodulatory systems, including the locus coeruleus (LC), ventral tegmental area (VTA), dorsal raphe nucleus (DRN), and basal forebrain, reach superficial cortical layers including L1 and modulate the activity states of cortical neurons (Aston-Jones and Cohen, 2005; Avery and Krichmar, 2017; Douglas and Martin, 2004). These inputs influence the dendritic integration properties of L2/3 and L5 pyramidal neurons, including their distal apical dendrites, and modulate the conditions governing overall neuronal excitability and synaptic plasticity (Lisman et al., 2012; London and Häusser, 2005; Stuart and Spruston, 2015).

In the present study, L1 is positioned not merely as an auxiliary input layer but as an important input layer that reflects CVMS-mediated neuromodulation in the operating conditions of the entire cortical column. In particular, based on Fundamental Property 3 in section “1.1 Fundamental properties of neurons,” changes in the amount of charge within spines associated with GNS-derived neuromodulatory inputs to L1 are considered to contribute to the activity conditions of neurons in the column even when those spines are located distally from the soma (Tsubo, 2021).

**(2) L2/3: Distributed Representation and γ-Band Candidate-Generation Layer**

L2/3 consists of local cortical circuits containing pyramidal neurons and diverse interneurons (Douglas and Martin, 2004; Harris and Shepherd, 2015; Markram et al., 2015). Distributed representations related to objects, spatial relationships, actions, values, sensory features, and other information are formed in TAC and PAC. In the DCEM model, recurrent interactions between DCEM and PAC/TAC are considered to generate numerous candidate groups in parallel as transient γ-band activity (Fries, 2005; Lundqvist et al., 2016, 2018; Spitzer and Haegens, 2017).

At this stage, each candidate group is assumed not yet to be synchronized at the CE level and to remain in a transient and fluid state. Local circuits in L2/3, including inhibitory interneurons, rapidly modulate activity in nearby neurons and participate in synchronization and competition among candidates (Fries, 2005; Helmstaedter et al., 2009; Singer, 1999).

Therefore, in the present model, when a new recurrent loop is formed, local inhibitory effects are considered to suppress previously active neighboring recurrent loops and facilitate switching to a new candidate state. In addition, L3 output provides a major excitatory input to L5 within the same column. Thus, L2/3 is positioned as a central layer involved in local information sharing, candidate generation, and competition among distributed contextual candidates (Douglas and Martin, 2004; Harris and Shepherd, 2015; Markram et al., 2015; Helmstaedter et al., 2009).

**(3) L5: Recurrent-Loop Formation Layer**

L5 contains large pyramidal neurons that integrate local cortical inputs and send widespread long-range projections to the thalamus and other cortical areas (Douglas and Martin, 2004; Harris and Shepherd, 2015; Wang, 2013). In the present study, as shown in Figure 1, L5 is positioned as the principal output layer forming γ-band and β-band recurrent loops. It is assumed that outputs are transmitted to distributed cortical networks through widespread axonal projections from the large number of large L5 pyramidal neurons present in each physical column.

Repeated outputs from L5 are considered to re-excite recurrent circuits between DCEM and PAC/TAC and continuously promote the generation and updating of new contextual candidate groups (Douglas et al., 1995; Wang, 2001; Tsubo, 2021). Thus, L5 is considered to function as the principal output layer that recursively maintains current context-related activity and transmits it to distributed cortical networks.

**(4) L6: Thalamic and TRN Control Layer**

As shown in Figure 2, L6 projects to the thalamus and thalamic reticular nucleus (TRN) and is involved in modulation of thalamic relay activity and cortico-thalamic information transmission (Griffiths et al., 2022; Halassa and Sherman, 2019; Sherman, 2016; Sherman and Guillery, 2006). In the present study, L6 is positioned as a layer that dynamically modulates thalamic inputs according to cortical activity states and controls cortico-thalamic interactions so that β-band recurrent loops can continue.

**FIGURE 2:**
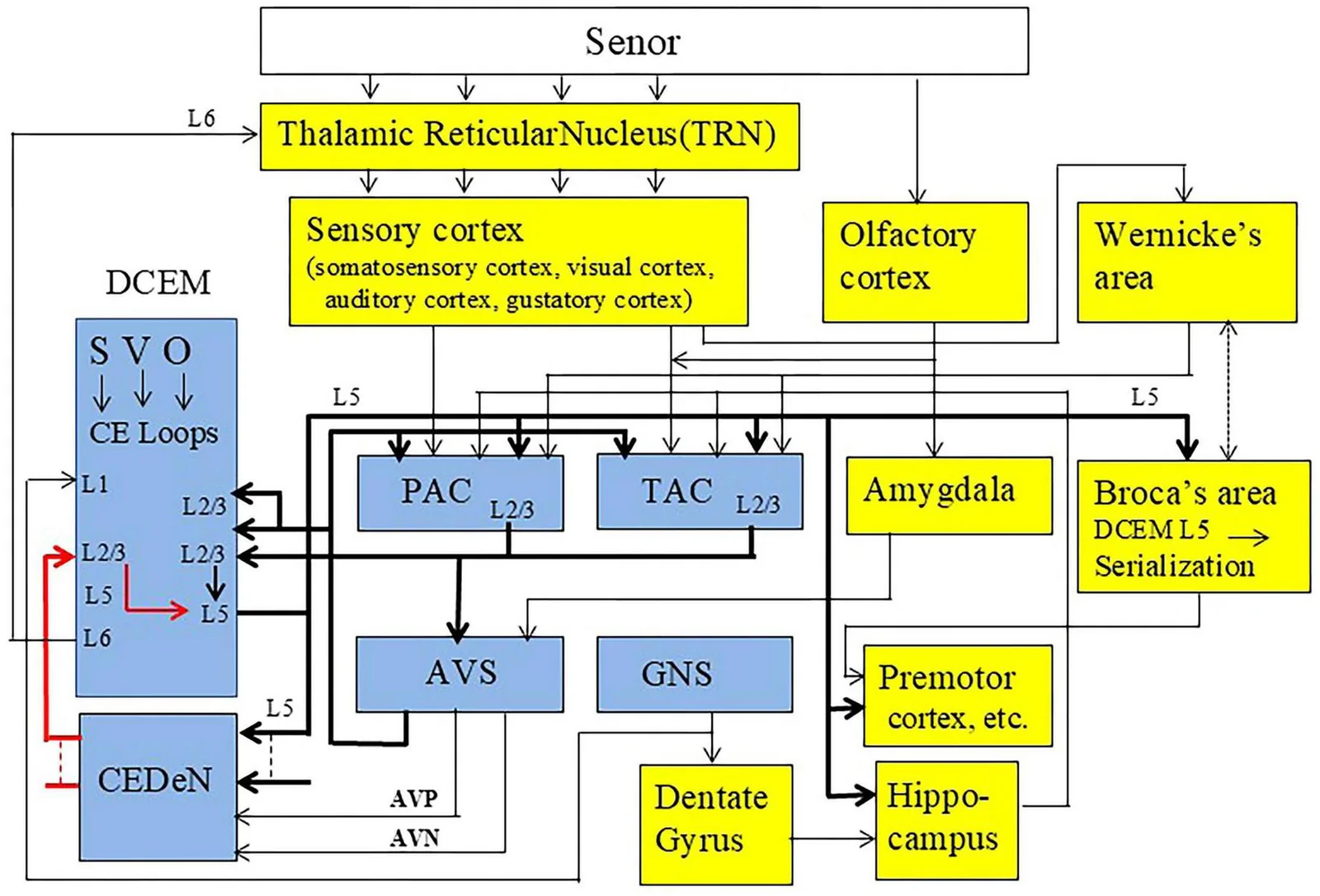
Contextual element (CE) loops and their associated functional pathways. Figure 1 illustrated the neural circuit connections that generate numerous asynchronous γ-band CE candidate loops driven by the AVS and β-band CE Loops initiated by synchronized feedback from the CEDeN. It shows the five major input pathways to PAC/TAC–AVS, sensory cortices, hippocampus, DCEM Layer 5 (L5), and Wernicke’s area–and their interconnections. Synchronized outputs from DCEM Layer 5 project to the hippocampal memory system, Broca’s area, premotor cortex, and other downstream output systems, thereby enabling contextual information established by β-band CE Loops to be decoded while maintaining temporal consistency. The GNS also projects to Layer 1 of each cortical column and to the hippocampal dentate gyrus (DG), coordinately modulating β-band CE Loops in the cortex and the subsequent initiation of episodic-memory formation. Furthermore, DCEM Layer 6 (L6) projects to the thalamic reticular nucleus (TRN) and is considered, through synchronized L6 outputs, to influence thalamic sensory gating by relatively suppressing sensory inputs with low relevance to the current CE Loops while facilitating the preferential transmission of sensory information relevant to the selected contextual state.

Taken together, conscious-state transitions in the DCEM model are understood as dynamic interlaminar processes involving interactions among candidate generation and maintenance by L2/3, neuromodulation through L1, recurrent output from L5, and cortico-thalamic control by L6 (Douglas and Martin, 2004; Halassa and Sherman, 2019; Sherman, 2016; Sherman and Guillery, 2006).

### 1.4 Functional organization of meaning groups in PAC and TAC

In the DCEM model, the context that guides behavior to fulfill internal desires is considered to potentially include functionally distinct information concerning who or what, does what, to what, in what state, when, where, why, and how. Accordingly, Subject, Verb, Object, Complement, When, Where, Why, and How (Attribute) are assumed as representative Contextual Element Categories (CE Categories), and the individual elements of context constituting each category are referred to as Contextual Elements (CEs).

The information corresponding to the specific contents of these CEs is assumed to exist in a distributed manner as numerous Meaning Groups formed through experience within the Association Cortices (AC). Each Meaning Group is considered to be a neuronal population representing a specific object, attribute, concept, action, or other information, and to retain information acquired through perception and experience as a functional unit (Eichenbaum, 2017; Fusi et al., 2016; Hickok and Poeppel, 2003; Rigotti et al., 2013).

In the present model, these Meaning Groups are functionally divided into two association cortices: the Perceptual Association Cortex (PAC) and the Temporal Association Cortex (TAC).

However, PAC and TAC do not represent strict anatomical subdivisions; rather, as shown in Table 1, they are functional classifications that primarily handle perceptual information and conceptual information, respectively, according to CE Category (Fedorenko and Blank, 2020; Goodale and Milner, 1992; Hickok and Poeppel, 2003; Rizzolatti and Matelli, 2003).

**TABLE 1:** Representative contextual information in PAC and TAC corresponding to each contextual element category (CE category).

CE category	Representative contents in PAC	Representative contents in TAC
Subject	•Persons and living things •Artificial objects and tools •Self and social entities	•Person names •Object names •Pronouns and noun phrases
Verb	•Observed actions and movements •Biological and physical events •Motor action representations	•Action verbs •State verbs •Auxiliary verb structures
Object	•Manipulated objects •Foods, books, vehicles, devices •Visual object representations	•Object names •Technical terms •Nominal expressions
Complement	•States and outcomes •Emotional and physical conditions •Goal or result representations	•Predicate complements •Adjectives •Complement expressions
When	•Temporal sequence of events •Episodic temporal context •Circadian and seasonal information	•Temporal words •Tense expressions •Time-related phrases
Where	•Spatial layouts •Object locations •Environmental maps	•Place names •Spatial prepositions •Location expressions
Why	•Motivation and intention •Rewards and avoidance context •Goal-directed information	•Causal expressions •Purpose clauses •Reason-related expressions
How (Attribute)	•Shape, color, size, texture •Motion, force, speed •Sensory attributes and methods	•Adverbs •Attribute words •Manner and descriptive expressions

PAC and TAC are not strict anatomical subdivisions, but rather functional classifications that primarily process perceptual information and conceptual/semantic information, respectively. In the DCEM model, Subject, Verb, Object, Complement, When, Where, Why, and How (Attribute) are defined as representative Contextual Element Categories (CE Categories) for constructing a context that guides behavior to satisfy desires, and the individual contextual elements assigned to each category are assumed to be Contextual Elements (CEs). This table presents representative examples of information corresponding to CE contents that is distributed and retained as Meaning Groups in PAC and TAC, and these types of information contribute to the formation of CE candidates through their interactions. The contents shown in this table are representative examples and do not indicate exclusive localization to specific brain regions or an exhaustive classification.

Meaning Groups in PAC represent diverse forms of information for directly perceiving the external world and the state of the self. Representative examples include identification of faces and persons, facial expressions and gaze direction, body posture, biological motion, hand movements, self-motion sensation, object shape, color, size, texture, and material, appearance of tools, landscapes, buildings, terrain, spatial arrangements, speech sounds and environmental sounds, music, odors, taste, touch, pain, temperature sensation, proprioception, emotional expressions, perception of danger, joint attention, social situations, and sensory memory cues, as well as other perceptible objects and states (Fedorenko and Blank, 2020; Goodale and Milner, 1992; Greve et al., 2018; Hickok and Poeppel, 2003; Rizzolatti and Matelli, 2003).

In contrast, Meaning Groups in TAC are considered to represent concepts and semantic knowledge abstracted from perceptual information. These include person concepts, names, social roles, body concepts, motor intentions, object concepts, categories, object functions, knowledge of tool use, place concepts, environmental knowledge, vocabulary, phonological representations, semantic concepts, food concepts, preferences, body-state concepts, health concepts, emotion concepts, value judgments, verb concepts, action concepts, procedural knowledge, motor plans, spatial concepts, temporal concepts, event sequences, causal relationships, logical structures, goals, intentions, plans, decision-making strategies, social norms, understanding of others, semantic memory, autobiographical knowledge, future prediction, and problem-solving strategies (Eichenbaum, 2017; Fedorenko and Blank, 2020; Hickok and Poeppel, 2003; Rigotti et al., 2013; Rizzolatti and Matelli, 2003).

Meaning Groups in PAC and TAC are not considered to function independently; rather, they continuously interact through extensive corticocortical connections and integrate perceptual and conceptual information in parallel (Douglas and Martin, 2004; Fedorenko and Blank, 2020; Goodale and Milner, 1992; Hickok and Poeppel, 2003). As a result, numerous Contextual Element (CE) candidates reflecting the current environment, past experience, and internal state are generated.

In the present study, these CE candidates are considered to undergo evaluation by the CVMS and then to be selected through CEDeNs and integrated within DCEM as β-band CE Loops to form the current contextual state.

### 1.5 Major input pathways determining contextual content in DCEM

The content of the context formed in DCEM is not considered to be fixed, but rather to change dynamically through interactions among diverse information sources entering DCEM and PAC/TAC. In the present study, as shown in Figure 2, five major input pathways to PAC/TAC that contribute to context formation are assumed: (1) AVS-derived input, (2) external sensory input, (3) hippocampal memory input, (4) recurrent feedback input from DCEM Layer 5, and (5) language-system input.

**(1) AVS-Derived Input Pathway**

The AVS includes an innate Proto-AVS present from birth and an experience-dependent AVS formed through postnatal experience and learning. In the present model, their outputs are assumed to project not only to DCEM but also to L2/3 of PAC/TAC, as shown in Figure 2. The AVS is also considered to update its internal value state while continuously reflecting the activity of DCEM and PAC/TAC.

The AVS is considered to reflect diverse internal demand states involved in biological maintenance and behavioral selection, including appetite, sexual desire, danger avoidance, social desires, and rewards expectation (Menon and Uddin, 2010; Padoa-Schioppa, 2011; Schultz, 2016). Consequently, in the present model, even in response to the same sensory input, food-related candidate groups may be preferentially formed during hunger, whereas candidate groups related to avoidance behavior may be preferentially formed under dangerous conditions.

**(2) External Sensory Input Pathway**

The most fundamental input pathway consists of external sensory inputs such as visual, auditory, and somatosensory information (Douglas and Martin, 2004; Harris and Shepherd, 2015). These inputs reach the association cortices through primary and higher-order sensory cortices and form distributed representations of object features, spatial location, movement relationships, temporal changes, and related information (Douglas and Martin, 2004; Fusi et al., 2016; Harris and Shepherd, 2015; Rigotti et al., 2013).

In the visual system in particular, the ventral pathway is primarily involved in information processing related to object recognition, whereas the dorsal pathway is primarily involved in information processing related to space and action (Goodale and Milner, 1992; Rizzolatti and Matelli, 2003). In the present model, this information is considered to be represented as coded Meaning Groups in L2/3 of PAC/TAC and to provide the basis for formation of new candidate groups (Fusi et al., 2016; Hickok and Poeppel, 2003; Rigotti et al., 2013). When new sensory input arrives, a corresponding new candidate group is formed, and the contextual content is assumed to be continuously updated.

**(3) Hippocampal Memory Input Pathway**

The hippocampus and medial temporal lobe system play important roles in the retrieval of past episodic memories and related information (Eichenbaum, 2017; Hainmueller and Bartos, 2020). In the present model, depending on current sensory input and the contextual state of DCEM, related memories in the hippocampus are considered to be reactivated through the entorhinal cortex, thereby contributing to the formation of new candidate groups in PAC/TAC. In particular, the recurrent circuitry of CA3 is involved in pattern completion, which reconstructs related memory representations from partial input (Eichenbaum, 2017; Hainmueller and Bartos, 2020), and is considered to contribute to retrieval of memory information required for current context formation.

**(4) Recurrent Feedback Input Pathway from DCEM Layer 5**

Cortical L5 pyramidal neurons send long-range outputs to other cortical and subcortical regions (Douglas et al., 1995; Douglas and Martin, 2004; Harris and Shepherd, 2015; Wang, 2013). In the present model, repeated feedback inputs are assumed to be sent from large pyramidal neurons in DCEM L5 to L2/3 of PAC/TAC, and this pathway is positioned as an important recurrent pathway in contextual candidate formation.

Based on the fundamental neuronal properties proposed in the present study, this repeated feedback is considered to repeatedly activate latent information representations related to the current contextual state and to promote formation of new candidate groups (Tsubo, 2021).

**(5) Language-System Input Pathway**

Language information is processed by broad temporal and frontal cortical networks and interacts with semantic representations (Fedorenko and Blank, 2020; Hickok and Poeppel, 2003). In the present model, information generated by auditory language, visual language, and internal language is considered to interact with coded Meaning Groups and to enter the PAC/TAC network.

Furthermore, in the present model, language input is considered to provide new information to the existing contextual structure and to promote formation of new candidate groups. During conversation in particular, the current contextual state is continuously updated as the other person’s utterances are continuously input to PAC/TAC.

Taken together, context formation in DCEM is understood as a dynamic process established through interactions among AVS input, external sensory input, hippocampal memory input, recurrent feedback from DCEM, and language input.

### 1.6 Initiation and termination mechanisms of contextual element loops

As described in the preceding sections, the present model assumes that numerous γ-band candidate groups are formed in parallel in PAC/TAC through external sensory input, hippocampal memory input, AVS input, language input, and recurrent feedback input from DCEM L5. The association of γ-band activity with transient information representations and candidate neural activity is consistent with experimental studies (Fries, 2005; Lundqvist et al., 2016, 2018; Spitzer and Haegens, 2017).

In the present study, each candidate group is assumed to consist of multiple physical columns belonging to the same logical colum (same CE), with their outputs entering the corresponding integrative neuron. As shown in Figure 1, the transition from this γ-band candidate state to a synchronized β-band CE Loop is assumed to involve a specialized population of integrative neurons located within the thalamic MD nucleus that functionally links a logical column to the physical columns constituting it.

Previous studies have shown that the MD nucleus plays important roles in recurrent interactions with the prefrontal cortex and in modulation of cognitive functions (Bolkan et al., 2017; Halassa and Kastner, 2017; Halassa and Sherman, 2019; Sherman, 2016; Sherman and Guillery, 2006; Watanabe and Funahashi, 2012; Wolff and Halassa, 2024).

In the present study, this hypothetical integrative neuron is termed the Contextual Element Decision Neuron (CEDeN). A CEDeN is assumed to exist for each CE (logical column) and to integrate DCEM L5 inputs from multiple physical columns constituting the same CE with value evaluations by the AVS, which monitors the contents of PAC/TAC L2/3, thereby determining whether to initiate a CE Loop.

For the Mth CE belonging to the Lth category, the condition for initiation of a CE Loop by a CEDeN is approximately expressed by the following equation:

I = (ΣL5_LMi_ + ΣAVP_LMi_ – ΣAVN_LMi_) × GNS_LM_ ≧θ

- ΣL5_LMi_: input from DCEM Layer 5 of the ith physical column constituting the LM CE (Douglas and Martin, 2004; Harris and Shepherd, 2015; Markram et al., 2015; Wang, 2013)
- ΣAVP_LMi_: the ith positive evaluation of the LM CE by the AVS (Aston-Jones and Cohen, 2005; Menon and Uddin, 2010; Padoa-Schioppa, 2011; Schultz, 2016)
- ΣAVN_LMi_: the ith negative evaluation of the LM CE by the AVS (Aston-Jones and Cohen, 2005; Menon and Uddin, 2010; Padoa-Schioppa, 2011; Schultz, 2016)
- GNS_LM_: global gain-modulatory effect of the GNS on the LM CE (Aston-Jones and Cohen, 2005; Avery and Krichmar, 2017; Marder, 2012; McCormick and Bal, 1997; Sara, 2009; Spitzer and Haegens, 2017)
- θ: threshold for CE Loop initiation

Long-range outputs from L5 pyramidal neurons have an established anatomical basis (Douglas and Martin, 2004; Harris and Shepherd, 2015; Wang, 2013), and the neuromodulatory systems integrated as the GNS in the present model are also consistent with previous findings showing modulation of neural gain and network states (Aston-Jones and Cohen, 2005; Avery and Krichmar, 2017; Marder, 2012; Sara, 2009).

In contrast, the specific mechanism by which the CEDeN integrates these inputs according to the above equation is a hypothesis proposed in the present study.

When this initiation condition is satisfied, the CEDeN is assumed to send synchronized feedback to L2/3 of multiple physical columns constituting the Mth CE of the Lth category in DCEM. As a result, the activity of these physical columns becomes synchronized, followed by synchronization of L5 outputs, and synchronized β-band CE Loop activity is considered to begin across the entire LM logical CE.

The notion that γ-band and β-band activities are associated with different functional states is consistent with previous physiological studies (Fries, 2005; Lundqvist et al., 2016, 2018; Spitzer and Haegens, 2017); however, the mechanism by which the CEDeN produces a transition from a γ-band candidate loop to a β-band CE Loop is a hypothesis specific to the present model.

During the initial formation of a CE Loop, L5 activity entering the CEDeN from multiple physical columns is not sufficiently synchronized, and therefore ΣL5 is considered to be relatively small. At this stage, when positive evaluation by the AVS (ΣAVP) is sufficiently large, the integrated input I is assumed to exceed the threshold θ, allowing the CEDeN to initiate synchronized feedback output.

Once synchronized feedback from the CEDeN begins, L2/3 activity in the multiple physical columns constituting the same CE becomes synchronized, and L5 output becomes synchronized accordingly. As a result, synchronized L5 activity enters the CEDeN, and ΣL5 is considered to increase through temporal summation. This reduces the dependence of CE Loop maintenance on positive evaluation from the AVS and is assumed to allow recurrent synchronized activity to be maintained relatively stably.

Because the present study assumes, as one structural example, eight CE Categories each containing approximately 25 CEs, CE Loops corresponding to approximately 200 CEs can potentially be formed throughout DCEM. However, this number is not an anatomical constant but a structural assumption introduced to concretely describe the operation of the model.

In contrast, when the evaluation by the AVS monitoring PAC/TAC activity shifts in a negative direction, ΣAVN increases and the integrated input I falls below the threshold θ. As a result, synchronized feedback from the CEDeN ceases, the corresponding β-band synchronization can no longer be maintained, and the CE Loop is considered to disappear.

However, even while a CE Loop is maintained, external sensory, memory, and language information continues to enter PAC/TAC. Therefore, even when AVS output representing a major goal or desire continues, the specific information content appearing in PAC/TAC may change over time. As long as the AVS evaluation does not become negative in response to such changes, the current CE Loop may be maintained. In contrast, if the content of a particular CE no longer matches the current goal, that CE Loop is terminated through an increase in AVN, and new CE content is selected from the γ-band candidate groups being explored in parallel, leading to the formation of a new β-band CE Loop.

Taken together, in the present study, the formation, maintenance, updating, termination, and replacement of numerous CE Loops are considered to constitute the fundamental operations underlying conscious-state transitions. That is, a conscious state is understood not as a fixed representation but as a dynamic process in which numerous γ-band candidate loops are explored in parallel, a subset is selected through value evaluation by the AVS and input integration by CEDeNs, temporarily stabilized as synchronized β-band CE Loops, and subsequently replaced sequentially at the level of individual CE Loops in response to changes in internal and external conditions. The finding that γ-band and β-band activities have different functional roles is consistent with previous studies (Fries, 2005; Lundqvist et al., 2016, 2018; Spitzer and Haegens, 2017), whereas this CE-level mechanism of formation, maintenance, termination, and replacement is a hypothesis proposed by the DCEM model.

### 1.7 Functional consequences of CE loop formation

When a CE Loop is formed, its synchronized β-band activity is considered not only to maintain the selected contextual state but also to exert broad effects on information processing in multiple brain systems. In the present study, multiple functional changes are considered to occur in parallel with CE Loop formation. These functions can broadly be divided into those supporting maintenance of the CE Loop and those utilizing the formed CE Loop.

#### 1.7.1 Functions supporting CE Loop maintenance

**(1) Selective Sensory Control**

When a CE Loop is formed, the selectivity of sensory information related to the currently maintained contextual state is also considered to change. In the present study, as shown in Figure 2, projections from DCEM L6 to the thalamus and thalamic reticular nucleus (TRN) are considered to participate in this selective sensory control. Previous studies have shown that circuits comprising cortical L6, the thalamus, and TRN are involved in thalamocortical information transmission and sensory gating (Griffiths et al., 2022; Halassa and Sherman, 2019; Sherman, 2016; Sherman and Guillery, 2006).

In the present model, synchronized activity from DCEM L6 is considered to influence thalamic circuits including the TRN, thereby relatively suppressing sensory inputs with low relevance to the current CE Loop and prioritizing inputs with high relevance. For example, during reading, processing related to visual information may become predominant, whereas the influence of sensory inputs with low relevance to the context may decrease. Conversely, if AVS output related to danger becomes predominant, sensory information required for danger detection may exert a stronger influence on the formation of new candidates.

In the present study, this thalamic gating mechanism involving the TRN is considered to constitute one mechanism for selectively incorporating necessary new information while maintaining a stable contextual state (Griffiths et al., 2022; Halassa and Sherman, 2019; Sherman, 2016; Sherman and Guillery, 2006).

**(2) Enhancement of Latent Representation Exploration**

Even after formation of a CE Loop, recurrent feedback from DCEM Layer 5 to PAC/TAC L2/3 is assumed to continue in the present model. The involvement of recurrent cortical activity in persistent information representation is consistent with previous studies (Douglas et al., 1995; Wang, 2001).

In the present model, this repeated feedback is considered to reactivate latent representations within PAC/TAC related to the currently maintained CE and to continuously promote exploration of new γ-band candidate groups.

As a result, a dynamic state is considered to form in which the current contextual state is maintained as a β-band CE Loop while related knowledge, memories, values, and contextual candidates that may be required next continue to be explored in parallel as γ-band candidate activity.

For example, during conversation, related memories and language information are continuously reactivated in response to the other person’s utterances (Eichenbaum, 2017; Fedorenko and Blank, 2020; Hickok and Poeppel, 2003), and candidate groups for the next CE Loops are considered to be prepared in parallel.

#### 1.7.2 Functions utilizing formed CE loops

**(1) Preparation of Behavioral Output**

When a CE Loop is stabilized, behavioral preparation corresponding to that contextual state is also considered to proceed in parallel. The prefrontal cortex interacts with broad networks including motor-related cortices, basal ganglia, and thalamus, and is involved in behavioral selection and executive control (Miller and Cohen, 2001; Watanabe and Funahashi, 2012).

In the present model, depending on the selected CE state, behavioral systems required to realize the current context, including gaze shifts, postural control, speech preparation, and motor planning, are considered to be sequentially activated.

Accordingly, in the present study, a conscious state is positioned not merely as an internal representation but as a dynamic contextual state continuously linked to behavioral selection for realizing a goal or desire provided by the AVS (Miller and Cohen, 2001).

**(2) Preparation for Language Generation**

After CE Loop formation, the currently maintained contextual structure is considered to interact with frontal and temporal language networks (Fedorenko and Blank, 2020; Hickok and Poeppel, 2003). In the present model, the language-generation system centered on Broca’s area is assumed to temporally arrange the nonverbal contextual structure composed of multiple CEs and convert it into a linear linguistic sequence.

As a result, the formed contextual state can be expressed as speech, written sentences, or internal language.

Accordingly, in the present model, language is positioned not as constituting the conscious state itself, but as one output format that converts a contextual structure previously formed by CE Loops into a linguistic sequence. This is also consistent with the model’s position that the eight CE Categories used in the present study do not directly assign natural-language grammatical categories to the nervous system.

**(3) Preparation for Memory Consolidation**

The hippocampal-cortical system is widely supported as playing important roles in episodic-memory encoding, reactivation, and long-term memory formation (Eichenbaum, 2017; Hainmueller and Bartos, 2020; Yang et al., 2024). In the present model, when a CE Loop is stably maintained for a certain period, information corresponding to the contextual structure at that time is considered to enter the hippocampal memory system through the entorhinal cortex and to be utilized for episodic-memory formation.

Furthermore, the synchronized activity state maintained in DCEM is assumed to potentially provide a temporal framework for memory encoding by transmitting distributed contextual information to the hippocampal system while preserving temporal consistency.

Accordingly, the present model predicts that contextual states maintained for a certain period as stable CE Loops are more likely to proceed to memory formation, whereas γ-band candidate activity that is not selected and disappears rapidly is not consolidated into episodic memory to the same extent. This relationship is one of the experimentally testable hypotheses derived from the DCEM model.

Taken together, in the present study, CE Loop formation is positioned not merely as local β-band synchronized activity, but as a central dynamic mechanism that stabilizes the current contextual state while coordinating sensory gating, latent-representation exploration, behavioral preparation, language generation, and memory formation. Through interactions among these functions, contextual states that change over time are considered to be continuously linked to perception, behavior, language, and memory.

## 2 Functional organization based on the DCEM framework

Chapter 1 described the formation and maintenance of CE Loops by DCEM and the transition mechanism from γ-band candidate states to β-band stable states. In this chapter, known neuroscientific findings regarding episodic memory, real-time language processing, and learning are interpreted in an integrated manner from the perspective of this dynamic context-formation mechanism. Rather than treating episodic memory, real-time language processing, and learning as independent functions, we regard them as different functional aspects of a common neural mechanism involving the formation, maintenance, reconstruction, and long-term consolidation of CE Loops, and present the corresponding theoretical framework (Eichenbaum, 2017; Fedorenko and Blank, 2020; Fusi et al., 2016; Rigotti et al., 2013).

### 2.1 Episodic memory within the DCEM framework

Episodic memory reconstructs past events together with their temporal and spatial contexts, as shown in Figure 2 (Eichenbaum, 2017; Hainmueller and Bartos, 2020). The hippocampal dentate gyrus (DG) is involved in pattern separation of novel inputs, whereas the recurrent circuitry of CA3 is known to be involved in pattern completion, which reconstructs related memory representations from partial cues (Eichenbaum, 2017; Hainmueller and Bartos, 2020). Furthermore, interactions between the hippocampus and cerebral cortex play important roles in episodic-memory retrieval and long-term memory formation (Eichenbaum, 2017; Hainmueller and Bartos, 2020).

However, how these hippocampal functions are linked to the current contextual state that changes in real time remains insufficiently understood (Eichenbaum, 2017).

In the present study, these known hippocampal functions are interpreted in an integrated manner based on the DCEM framework. In DCEM, the formation and retrieval of episodic memory are understood not as the static storage and reproduction of memory information, but as processes that interact with the formation, maintenance, and reconstruction of CE Loops composed of Contextual Elements (CEs).

Memory traces related to current sensory input, internal state, or language input are considered to be reactivated through the hippocampal system and to contribute to the formation of new candidate representations in PAC/TAC (Eichenbaum, 2017; Hainmueller and Bartos, 2020). In the present model, these memory-derived representations also become part of the γ-band candidate groups, like other candidate information, and contents selected through value evaluation by the AVS and selection by CEDeNs are stabilized as β-band CE Loops and incorporated into the current contextual state as past events.

Furthermore, in the present model, recurrent feedback from DCEM Layer 5 to PAC/TAC Layer 2/3 is assumed to continuously reactivate latent representations related to the content of the currently maintained CE. The involvement of recurrent cortical activity in persistent information representation is consistent with previous studies (Douglas et al., 1995; Wang, 2001). Therefore, episodic-memory retrieval can be understood not as a simple replay of once-stored information, but as a dynamic process in which related representations are explored and reconstructed according to the current contextual state.

The consolidation of episodic memory can also be interpreted in relation to known neurophysiological findings within the DCEM framework. Broad neuromodulatory systems regulate cortical activity states and the conditions for hippocampal memory formation (Aston-Jones and Cohen, 2005; Avery and Krichmar, 2017; McCormick and Bal, 1997; Sara, 2009). In addition, inputs to the distal dendrites of cortical pyramidal neurons affect their integrative properties and plasticity (Lisman et al., 2012; London and Häusser, 2005; Major et al., 2013; Stuart and Spruston, 2015).

In the present model, when a β-band CE Loop is stably maintained for a certain period under such a neuromodulatory state, information corresponding to the contextual content at that time is considered to enter the hippocampal system and become more readily utilized for episodic-memory formation in hippocampal circuits including the DG (Eichenbaum, 2017; Hainmueller and Bartos, 2020; Yang et al., 2024). Therefore, the GNS may function as a common modulatory mechanism affecting both the conditions for CE Loop formation in the cortex and the conditions for memory encoding in the hippocampus (Aston-Jones and Cohen, 2005; Avery and Krichmar, 2017; Hainmueller and Bartos, 2020; Yang et al., 2024).

Furthermore, it is widely known that Ca^2 +^ influx through NMDA receptors, CaMKII activation, and structural and functional plasticity of dendritic spines are important for LTP formation (Kasai et al., 2010; Lisman et al., 2012; Magee and Johnston, 2005). In the present study, we consider the possibility that, through interactions between these known synaptic plasticity mechanisms and the mechanism of charge retention within spines proposed in our previous studies (Tsubo, 2021), some synaptic states associated with sufficiently stabilized CE Loops become fixed over the long term.

Taken together, in the present study, episodic memory is positioned not as a simple storage-and-retrieval system independent of DCEM, but as a dynamic process in which the current contextual state formed by sufficiently stabilized CE Loops is consolidated as memory through interactions with the hippocampal system, and information reactivated from the hippocampus during subsequent retrieval is again incorporated into the context-formation process of DCEM.

### 2.2 Real-time language processing within the DCEM framework

Language is a cognitive function that enables advanced information communication and expression of thought in humans. In current language neuroscience, language comprehension and language generation are considered to be realized not by a single localized area, but through interactions among broad networks including the frontal, temporal, and parietal lobes (Fedorenko and Blank, 2020; Hickok and Poeppel, 2003).

However, the integrated neural mechanism by which external input, memory retrieval, value evaluation, and generation of the next speech candidates are realized simultaneously and in parallel while the current conversational context is maintained remains insufficiently understood.

In the present study, this real-time language processing is interpreted in an integrated manner through the context-formation mechanism of DCEM. In DCEM, language itself is not positioned as forming the conscious state, but as the result of converting an already formed nonverbal CE Loop structure into a linear linguistic sequence with temporal order.

Regarding language input, as shown in Figure 2, auditory and visual language are considered to be converted through language-related networks into phonological, lexical, and semantic representations, which interact with distributed semantic representations in PAC/TAC (Fedorenko and Blank, 2020; Hickok and Poeppel, 2003). In the present model, these language-derived representations, like external sensory input and memory input, contribute to the formation of numerous γ-band candidate groups, some of which are assumed to be selected through AVS evaluation and CEDeN-mediated selection and stabilized as β-band CE Loops.

Regarding language output, the formed contextual structure is considered to be transmitted to frontal and temporal language networks and converted into a temporally ordered linguistic sequence (Fedorenko and Blank, 2020; Hickok and Poeppel, 2003).

In the present model, the language-generation network including Broca’s area is assumed to arrange the relational structures among multiple CEs that are maintained simultaneously and in parallel within DCEM according to temporal order and to serialize them through syntactic structuring and lexical selection into temporally ordered speech sequences.

In this process, broad output from DCEM Layer 5 is considered to function as one pathway that transmits the currently formed contextual state to the relevant cortical networks. The fact that cortical L5 pyramidal neurons mediate long-range cortical and subcortical projections is consistent with existing anatomical findings (Douglas and Martin, 2004; Harris and Shepherd, 2015; Wang, 2013), whereas the specific mechanism of contextual output from DCEM to the language network is a hypothesis proposed by the present model.

In DCEM, the continuity of real-time conversation is also explained by this dynamic context-maintenance mechanism. During conversation, related memories, values, behavioral candidates, and language candidates are activated in parallel in PAC/TAC according to the other person’s utterances (Eichenbaum, 2017; Fedorenko and Blank, 2020; Hickok and Poeppel, 2003; Rigotti et al., 2013).

In the present model, the overall conversational context is considered to be maintained and updated as appropriate CE contents are sequentially selected from among these candidates and stabilized as individual CE Loops.

Furthermore, recurrent feedback from DCEM Layer 5 (L5) to PAC/TAC is assumed to continuously reactivate latent representations related to the current contextual state, thereby promoting the exploration of new candidate representations.

Therefore, while understanding the current utterance, it is considered possible to prepare related memories, value evaluations, candidates for the next utterance, and behavioral candidates in parallel.

Taken together, in the present study, language is positioned not as an independent thought-generation system, but as a bidirectional input-output mechanism that converts the multidimensional contextual structure formed and maintained by DCEM into a linear representation with temporal order, while also re-entering externally obtained linguistic information into the context-formation system.

### 2.3 Learning within the DCEM framework

Learning is a process of neural plasticity through which new information is integrated into existing knowledge systems and organized into a form that can be reused in the future. Long-term potentiation (LTP), synaptic plasticity, and hippocampal-cortical interactions are important neural bases of learning and memory formation (Eichenbaum, 2017; Kasai et al., 2010; Lisman et al., 2012). In addition, Ca^2 +^ influx through NMDA receptors, CaMKII activation, and structural changes in dendritic spines play important roles in synaptic plasticity (Kasai et al., 2010; Lisman et al., 2012; Magee and Johnston, 2005).

However, the integrated neural mechanism by which new knowledge becomes linked to existing knowledge structures and develops into flexible concept formation and adaptive behavior within a limited period remains insufficiently understood.

In the present study, learning is interpreted from the perspective of the dynamic context-formation mechanism of DCEM. In DCEM, learning is understood not as an independent information-storage process, but as a process in which existing CE structures are reused while relationships with new CE contents are formed and reorganized, and some of these are fixed as long-term neural changes.

External sensory input, memory input, and language input form numerous candidate representations in PAC/TAC (Douglas and Martin, 2004; Eichenbaum, 2017; Fedorenko and Blank, 2020; Hickok and Poeppel, 2003; Rigotti et al., 2013). In the present model, these are explored as γ-band candidate groups, and contents selected through AVS evaluation and CEDeN-mediated selection are stabilized as β-band CE Loops, thereby forming new contextual states. Furthermore, recurrent feedback from DCEM L5 to PAC/TAC L2/3 is assumed to continuously reactivate latent representations related to the currently maintained CE state. The involvement of cortical recurrent circuits in persistent activity and information representation is consistent with previous studies (Douglas et al., 1995; Wang, 2001). In the present model, through this recurrent feedback and the resulting exploration of candidate representations, new candidate information is considered to be incorporated into more coherent contextual structures while repeatedly being associated with existing CE structures.

Such reuse of existing structures is also consistent with the human ability to rapidly understand new concepts by using metaphors and analogies. For example, in the metaphorical understanding that “electric current is like water flow,” part of an already formed relational structure concerning “flow,” “resistance,” and “pressure difference” can be reused in understanding a new concept. In the present model, such analogical learning is interpreted as partial reuse of existing CE structures and formation of relationships with new CE contents (Fusi et al., 2016; Rigotti et al., 2013).

For long-term consolidation of learning, both known neural plasticity mechanisms and the authors’ biophysical hypotheses are considered. Ca^2 +^ influx through NMDA receptors and CaMKII-dependent LTP formation are known mechanisms of synaptic plasticity (Kasai et al., 2010; Lisman et al., 2012; Magee and Johnston, 2005). Furthermore, in our previous studies, we proposed the possibility that charge retained in spines contributes to the integrative state of neurons (Tsubo, 2021, 2023, 2024). In DCEM, through these effects, synaptic states associated with repeatedly stabilized CE Loops are considered to change over the long term and to become more readily reusable in future context formation.

In addition, the broad neuromodulatory state produced by the GNS may affect both the conditions for CE Loop formation in the cortex and the conditions for memory encoding in the hippocampal system (Aston-Jones and Cohen, 2005; Avery and Krichmar, 2017; Hainmueller and Bartos, 2020; Yang et al., 2024). Therefore, in the present model, learning and episodic-memory formation are understood not as completely independent processes, but as mutually related processes mediated through context formation and neural plasticity.

As described above, in this chapter, episodic memory, real-time language processing, and learning are presented in an integrated manner within a single theoretical framework of the DCEM model.

However, for such an integrated framework to be valid, its correspondence with the underlying biophysical mechanisms at the neuronal level must be clear.

### 2.4 Functional relationship between CVMS and DCEM in conscious-state transitions

In the present study, the Context Valuation and Modulatory System (CVMS) is positioned as an integrated functional system for valuation and neuromodulation, consisting of the Affective Valuation System (AVS) and the Global Neuromodulatory System (GNS). The AVS provides the direction of what the organism seeks, avoids, and evaluates as valuable, whereas the GNS is considered to modulate the gain, excitability, and persistence conditions of overall neural activity (Aston-Jones and Cohen, 2005; Avery and Krichmar, 2017; Menon and Uddin, 2010; Padoa-Schioppa, 2011; Schultz, 2016).

In the present framework, the AVS may be interpreted as one important neural function constituting the “mind” in a biological sense. That is, the AVS provides direction based on internally generated desires and values concerning what the organism seeks, what it avoids, and what it regards as important, while the GNS modulates the strength and persistence conditions of their effects on neural activity. In contrast, DCEM forms, maintains, and updates contextual structures under these valuation and modulatory conditions and links them to behavior.

In the present study, conscious-state transitions are considered to arise through the formation, maintenance, termination, and replacement of individual CE Loops within DCEM under the value-based drive and neuromodulatory conditions provided by the CVMS.

In the present study, the AVS is not assumed to be a fixed system that is fully developed at birth. At birth, the Proto-AVS, which represents innate values related to survival and preservation of the species, is considered to play the primary role. These include biologically fundamental values such as hunger, thirst, avoidance of danger and pain, and maintenance of safety (Padoa-Schioppa, 2011; Schultz, 2016). These innate values are considered to constitute fundamental factors driving context formation during the early postnatal period.

In humans, however, the content of value evaluation is considered to be continuously modified and expanded through experience, memory, and learning (Eichenbaum, 2017; Kasai et al., 2010; Padoa-Schioppa, 2011). In the present study, the value-evaluation function formed through such experience is positioned as the experience-dependent higher-order AVS. Therefore, the values and desires that drive context formation in adults are considered to reflect both elements of the innate Proto-AVS and value evaluations acquired through lifelong experience.

The functional state of the GNS may also change depending on experience and the history of neural activity (Aston-Jones and Cohen, 2005; Avery and Krichmar, 2017; Marder, 2012; Sara, 2009). As a result, the gain, excitability, and persistence conditions under which AVS-guided contextual selection and CE Loop formation occur may also change in a state-dependent manner. However, the detailed mechanisms by which specific developmental or social experiences form specific psychological characteristics are outside the scope of the present study.

Accordingly, the present framework does not propose a theory explaining psychological development in general. The purpose of distinguishing the innate Proto-AVS from the experience-dependent higher-order AVS in this section is limited to showing that the value signals driving DCEM are formed not only by innate factors but also through experience.

In the present study, DCEM is considered to dynamically select, form, maintain, and update contextual structures on the basis of these continuously changing AVS value signals and GNS-mediated neuromodulatory conditions. Accordingly, the CVMS is positioned as providing the direction and activity conditions for conscious-state transitions, whereas DCEM is positioned as the mechanism that dynamically forms specific contextual content under those conditions.

From this perspective, when the term “Mind” is used, it is used in a limited sense as a functional expression of the internal drive state formed by the organism’s desires, value evaluations, and neuromodulation, rather than as an independent psychological entity. In contrast, what the present study directly seeks to explain is the conscious-state transitions that arise through the formation, maintenance, updating, and termination of CE Loops within DCEM on the basis of these driving conditions.

In the next chapter, the fundamental properties of neurons underlying DCEM are described, focusing on a series of biophysical studies previously conducted by the authors (Tsubo and Kurokawa, 2018; Tsubo, 2021, 2023, 2024). This demonstrates that the DCEM model is a theoretical framework derived stepwise from analysis of biophysical properties at the neuronal level, through cortical columns and cortico-thalamic circuits, to a system-level mechanism of context formation.

## 3 Biophysical properties supporting DCEM dynamics

Chapters 1 and 2 described the mechanisms of context formation, conscious-state transitions, memory, language, and learning based on the DCEM framework. In this chapter, the biophysical properties of neurons that support these dynamic processes are described. First, based on our study demonstrating a mechanism of rapid action potential propagation (Tsubo and Kurokawa, 2018), we describe the study in which charge retention, temporal integration, and manifestation of latent information in dendritic spines were analyzed by considering the extremely high relative permittivity of intracellular fluid derived from that study (Tsubo, 2021). We then describe the computational functions of neurons for excitatory and inhibitory inputs that were derived on the basis of these properties (Tsubo, 2023, 2024).

### 3.1 Biophysical basis of rapid action potential propagation

It has long been reported that in thick myelinated mammalian nerve fibers, conduction velocity increases with axon diameter and can exceed approximately 100 m/s (Hursh, 1939). In addition, the localization and density of Na^+^ channels in myelinated axons play important roles in rapid action potential propagation (Ritchie and Rogart, 1977). In contrast, the diffusion coefficients of ions such as Na^+^ and Ca^2 +^ in aqueous solution are generally on the order of 10^−9^ m^2^/s, and rapid signal transmission along the longitudinal axis of an axon cannot be explained by simple diffusion of individual ions (Hille, 2001).

On the basis of these conditions, in Tsubo and Kurokawa (2018), we did not treat intracellular fluid simply as a resistive conductor, but considered its dielectric properties and quantitatively analyzed the possible contribution of the capacitive component and displacement current formed in the axial direction to action potential propagation.

The displacement current I is expressed by the following relationship.


I=CdV/dt

In the present model, this relationship is used to analyze both the displacement current formed in the axial direction and the capacitive current through the axonal membrane (Tsubo and Kurokawa, 2018).

In the equation, dV/dt represents the rising rate of the action potential. At sites of action potential generation, particularly the nodes of Ranvier, voltage-gated Na^+^ channels are known to be localized at high density (12,000 channels/μm2) (Ritchie and Rogart, 1977), and rapid Na^+^ influx within a short period produces a steep change in membrane potential. This steep voltage change produces a large dV/dt.

Although γ-band and β-band loop activities in the brain repeat at relatively long periods, the rising phase of each individual action potential constituting these activities is extremely steep. Therefore, the principal factor determining the magnitude of displacement current in the present analysis is not the oscillation period itself, but dV/dt of each individual action potential (Tsubo and Kurokawa, 2018).

However, even when dV/dt is the same, reducing the capacitive current through the axonal membrane and relatively increasing the capacitive current along the longitudinal axis of the axon are advantageous for rapid conduction. In our equivalent-circuit analysis, this condition was examined in terms of the relationship between membrane capacitance Cm and axial capacitance Ca (Tsubo and Kurokawa, 2018).

#### (1) Reduction of Membrane Leakage Displacement Current by Decreasing Axonal Membrane Capacitance

The membrane capacitance Cm per unit length of a cylindrical axon is determined by the permittivity of the axonal membrane and its inner and outer diameters (Tsubo and Kurokawa, 2018).


Cm=2⁢π⁢ε/ln⁢(Do/Di)

The permittivity ε of the axonal membrane is smaller than that of intracellular fluid, and in myelinated axons, the thick myelin sheath further markedly reduces the effective membrane capacitance Cm. In our analysis, we calculated that, when an axon with a diameter of 20 μm has a myelin sheath approximately 4 μm thick, membrane capacitance Cm decreases to less than 1% of its original value, demonstrating that the displacement current through membrane capacitance Cm is greatly reduced (Tsubo and Kurokawa, 2018).

#### (2) Increase in Axial Displacement Current by Increasing Axial Capacitance

In our equivalent circuit, axial capacitance Ca was defined in relation to the permittivity of the axoplasmic fluid, axonal cross-sectional area, and signal propagation distance as follows (Tsubo and Kurokawa, 2018).


Ca=2⁢ε⁢S/d=2⁢ε⁢ε0⁢Sa/d

Here, 2 indicates the directionality in which the electric field spreads, ε is the permittivity of the axoplasmic fluid, ε_0_ is the vacuum permittivity, ε_*a*_ is the relative permittivity of the axoplasmic fluid, (S) is the axonal cross-sectional area, and (d) is the distance from the site of generation to the observation point (Tsubo and Kurokawa, 2018).

In (Tsubo and Kurokawa, 2018), a myelinated axon with a diameter of 20 μm and an action potential rise time of 125 μs was analyzed, and the relative permittivity required for the axoplasmic fluid was calculated under the condition that a conduction velocity of approximately 120 m/s, including the action-potential relay time, was achieved.

As a result, an extremely large relative permittivity of approximately 10^6^ was obtained. Experimental studies of dielectric properties have also reported that biological tissues exhibit extremely large permittivity in the low-frequency range (Gabriel et al., 1996a,b).

Based on the consistency between these measured values and those obtained from the equivalent-circuit analysis, we proposed that axial capacitance resulting from the high dielectric properties of intracellular fluid may contribute to rapid action potential conduction (Tsubo and Kurokawa, 2018).

Accordingly, in the present model, the combination of a large dV/dt produced by the steep rise of the action potential, low membrane capacitance resulting from myelination, and axial capacitance generated by the high dielectric properties of intracellular fluid and the axonal cross-sectional area is considered to constitute a biophysical condition enabling rapid and low-attenuation signal propagation (Tsubo and Kurokawa, 2018).

In conventional cable models, capacitance is generally incorporated in the membrane direction, whereas a capacitance component along the direction of signal propagation is not explicitly included. Introduction of the axial capacitance therefore requires an extended circuit formulation and analysis using the corresponding partial differential equations (Tsubo and Kurokawa, 2018).

This relationship also provides a biophysical basis for understanding the functional significance of axonal diameter and postnatal myelination in rapid signal propagation.

In addition, the analytical result indicating the high relative permittivity of axoplasmic fluid provided the basis for applying the same electrical properties of intracellular fluid to dendritic and spine fluids, as described in the next section, and subsequently developed into analyses of spine charge retention and neuronal computational functions (Tsubo and Kurokawa, 2018; Tsubo, 2021, 2023, 2024).

### 3.2 Charge-retention properties of dendritic spines and extended synaptic integration

One of the essential functions of neurons is to integrate numerous synaptic inputs on dendrites and ultimately link them to action potential firing (London and Häusser, 2005; Magee and Johnston, 2005; Major et al., 2013; Stuart and Spruston, 2015). It has traditionally been known that temporal and spatial summation of multiple excitatory postsynaptic potentials (EPSPs) occurring almost simultaneously constitutes a fundamental mechanism of synaptic integration (London and Häusser, 2005; Magee and Johnston, 2005; Stuart and Spruston, 2015).

In contrast, in higher-order information processing such as conversation and reading, synaptic integration must also be established by relating inputs within the same neuron even when temporal differences exist between those inputs. It has long been expected that dendritic spines retaining charge may contribute to the establishment of such synaptic integration. However, a biophysical mechanism demonstrating how charge retained in individual spines could contribute to synaptic integration at the level of the entire neuron had not been established. Therefore, in Tsubo (2021), the high dielectric properties of intracellular fluid analyzed in the preceding study were also applied to dendritic fluid and spine-neck fluid, and the influence of charge retained within spines on the electrical state of the entire neuron was analyzed using an equivalent capacitive circuit.

In this analysis, the effect of the amount of charge Q retained within a spine on the membrane potential of the entire cell membrane was analyzed using an equivalent capacitive circuit. Because details of the analysis were presented in Tsubo (2021), only the points necessary for the present study are described here. Because a dielectric can accumulate charge through polarization, it can be treated as a capacitance. Based on this concept, an equivalent circuit consisting of spine membrane capacitance Csm, spine-neck capacitance Cn, dendritic capacitance (Cd), and total membrane capacitance ΣCm of the entire cell membrane was constructed (Tsubo, 2021).

Because spine-neck capacitance Cn, dendritic capacitance Cd, and total membrane capacitance ΣCm are present in series from the charge Q retained within the spine to the extracellular space, the equivalent series capacitance Cs is expressed by the following equation (Tsubo, 2021).


1/Cs=1/Cn+1/Cd+1/Σ⁢Cm

Furthermore, based on the fundamental relationship among voltage V, capacitance C, and charge Q (Q=CV), the increase in potential represented by the voltage V between the spine-retained charge Q and the extracellular space is given by the following equation.


V=Q/(Csm+Cs)

Because spine membrane capacitance Csm is sufficiently small compared with the other capacitance components, when the pathway through spine membrane capacitance Csm is approximately neglected, the relationship among voltage V, charge Q, and capacitance Cs becomes:


V=Q/Cs=Q⁢(1/Cn+1/Cd+1/Σ⁢Cm)

Furthermore, when n spines retaining approximately the same amount of charge Q are connected in parallel on the same dendrite, the relationship is expressed as follows (Tsubo, 2021).


V=nQ⁢(1/nCn+1/Cd+1/Σ⁢Cm)

=Q/Cn+nQ/Cd+nQ/Σ⁢Cm=V⁢1+V⁢2+V⁢3

This equation indicates that V3 is the total amount of intracellular charge nQ divided by the total membrane capacitance ΣCm. Here, when the resting membrane potential is Vs(−65 mV) and the firing threshold of Na^+^ channels is Vna(−50 mV), the condition for action potential generation is given by the following equation (Tsubo, 2021).


V3=nQ/Σ⁢Cm≧θ=Vna-Vs=15⁢mV

This equation indicates that when the amount of charge retained by n spines exceeds a certain level relative to the total membrane capacitance ΣCm, it can contribute to action potential firing. However, in practice, if charge has already accumulated in the spines, the action potential will occur at the timing of the next EPSP.

The following characteristics are derived from this analysis (Tsubo, 2021).

(1) In the present model, charge retained within the cell is considered to contribute to the electrical state of the entire cell irrespective of its location. However, because excess charge injected into a dendrite is regulated by ion channels, pumps, and other mechanisms to return the membrane potential toward the resting potential, charge retained within local compartments such as spines may contribute to integration over a relatively long time window (Tsubo, 2021). Therefore, charge retained in spines located on distal dendrites of Layer 5 pyramidal neurons, for example near Layer 1, is also considered capable of contributing to the firing conditions of the entire cell while it is retained (Tsubo, 2021).

(2) Because of diffusion restriction by the spine neck, charge Q within the spine can be retained for a relatively long period. The retention time is considered to depend on spine morphology and neck structure (Kasai et al., 2010; Magee and Johnston, 2005; Tsubo, 2021). Ca^2 +^ ions and Ca^2 +^-activated proteins such as CaMKII are also known to be retained or remain active within spines and to play important roles in the persistence of synaptic states (Lisman et al., 2012). This relatively long retention of charge Q allows preceding excitatory inputs to remain effective when subsequent inputs arrive after a temporal interval, thereby extending the time window over which synaptic inputs can contribute to neuronal integration (Tsubo, 2021).

(3) When new excitatory input is added to a spine in which retained charge remains, the retained state can be further enhanced. We proposed that, because inputs are repeated through recurrent loops, the number of spines retaining charge increases, thereby allowing neurons that had not previously reached firing conditions to sequentially transition into an activatable state (Tsubo, 2021).

(4) In a neuron with m input-capable spines, if n or more inputs are assumed to contribute to the firing condition, the theoretical number of input combinations increases combinatorially (Tsubo, 2021). However, this represents a theoretical upper limit, and actual neuronal circuits are constrained by dendritic structure, input correlations, spine arrangement, network connectivity, and other factors.

Nevertheless, this analysis indicates that a single neuron possesses the potential ability to selectively integrate numerous different combinations of inputs, and in the present study this is positioned as Fundamental Property 1 in section “1.1 Fundamental properties of neurons.” In particular, this property is related to one of the biophysical bases by which Layer 2/3 pyramidal neurons in DCEM and PAC/TAC can form different output states in response to diverse combinations of contextual information (Tsubo, 2021).

### 3.3 Computational functions of neurons

Neurons function as nonlinear integrative elements that integrate excitatory and inhibitory inputs spatiotemporally and generate an action potential when the resulting membrane potential exceeds the firing threshold (Douglas and Martin, 2004; Harris and Shepherd, 2015; London and Häusser, 2005; Major et al., 2013; Stuart and Spruston, 2015).

In our analysis described in the preceding section (Tsubo, 2021), the condition for action potential firing was addressed primarily from the relationship between the amount of positive charge retained within the cell and the total membrane capacitance. In particular, as described in section “3.2 Charge-retention properties of dendritic spines and extended synaptic integration,” charge retained within dendritic spines can persist for a relatively long period and thereby allow excitatory inputs arriving at different times to contribute to the same integration process. In subsequent studies, this framework was extended to inhibitory inputs, and the positive electrical effects of excitatory inputs and negative effects of inhibitory inputs were expressed within the same computational framework (Tsubo, 2023, 2024).

In the present paper, the effect of membrane-potential reduction produced by inhibitory input is represented as an equivalent negative charge term, ΣQCl. This term primarily represents the inhibitory effect of Cl^–^ influx while excess negative charge is present relative to the resting state, and decreases as the membrane state returns toward the resting condition. Hyperpolarizing effects such as K^+^ efflux are also included as equivalent inhibitory effects (Tsubo, 2023, 2024).

In this case, the condition for action potential generation is expressed by the following equation.


(ΣQ-ΣQ)Cl/ΣCm≧θ

Whereas many excitatory synaptic inputs are formed on dendritic spines, inhibitory synapses are distributed across multiple sites including dendrites, the soma, and some spines (Feldman, 2012; Harris and Shepherd, 2015; Kasai et al., 2010; London and Häusser, 2005; Magee and Johnston, 2005). Therefore, when considering the effective charge described above, both the relatively long retention of charge within spines described in section “3.2 Charge-retention properties of dendritic spines and extended synaptic integration and the temporal and spatial distributions of inhibitory effects must be considered (Tsubo, 2021, 2023, 2024).

From this action-potential generation condition, we derived the following functional characteristics of neuronal circuits (Tsubo, 2023, 2024).

#### 3.3.1 Logical computational functions

We proposed that, through appropriate combinations of excitatory and inhibitory inputs, neurons and neuronal circuits can construct threshold-type logical operations corresponding to AND, OR, NAND, NOR, and related operations, and that combining multiple elements can realize more complex logical functions including XOR (Tsubo, 2023, 2024).

This does not mean that individual neurons are equated with fixed digital gates; rather, it means that diverse condition-dependent computations are possible through combinations of excitatory input, inhibitory input, retained charge, and firing threshold (Tsubo, 2021, 2023, 2024).

#### 3.3.2 Encoding and decoding of information

Information in the cerebral cortex is represented as distributed activity patterns across neuronal populations (Fusi et al., 2016; Rigotti et al., 2013). In the present model, selective threshold operations based on combinations of excitatory and inhibitory inputs are considered potentially usable for reading out such distributed codes (Tsubo, 2023, 2024).

This property is considered to provide one of the biophysical bases by which downstream neuronal circuits can read out, as specific combinations, CE information distributed and retained across multiple physical columns in the DCEM model.

#### 3.3.3 Mechanism for termination of recurrent loops

Inhibitory neuronal circuits are important for selectively terminating or switching persistent recurrent activity. Local and lateral inhibitory connections exist within the cortex (Helmstaedter et al., 2009), and in the present model, when a new recurrent loop is formed, inhibitory effects on neighboring columns are considered to terminate some of the previous γ-band candidate loops and replace them with new candidate states.

For β-band CE Loops, as described in the preceding chapter, when negative evaluation from the AVS causes the integrated input to the CEDeN to fall below the threshold, synchronized feedback is assumed to stop and the corresponding CE Loop to terminate. This CEDeN-dependent termination mechanism is a hypothesis specific to the DCEM model and is distinguished from replacement of γ candidate loops through local inhibition.

#### 3.3.4 Information retention through β-band synchronization

Previous studies have reported that β-band activity is related to maintenance and reactivation of the current internal state or selected information (Lundqvist et al., 2016, 2018; Spitzer and Haegens, 2017).

In the DCEM model, activity in multiple physical columns within the same CE synchronized by the CEDeN forms a β-band CE Loop, and its synchronized L5 outputs are repeatedly sent to PAC/TAC and the CEDeN, thereby allowing selected contextual information to be stably retained for a certain period. Furthermore, this synchronization ensures temporal consistency of CE information distributed and coded across multiple physical columns and is considered to stabilize the input conditions for decoding in downstream circuits (Tsubo, 2023, 2024).

### 3.4 Threshold model of the contextual element decision neuron (CEDeN)

Figure 3 presents a conceptual diagram of the Contextual Element Decision Neuron (CEDeN), which is hypothesized to be responsible for the initiation, maintenance, and termination of CE Loops. The MD nucleus is known to play important roles in recurrent interactions with the prefrontal cortex, information selection, and cognitive control (Griffiths et al., 2022; Halassa and Kastner, 2017; Halassa and Sherman, 2019; Sherman, 2016; Watanabe and Funahashi, 2012; Wolff and Halassa, 2024). In the present study, based on this known cortico-MD interaction, we hypothesize that a threshold-type integrative neuron or functional neuronal population exists within the MD nucleus for each Contextual Element (CE), integrating inputs from L5 of the corresponding multiple DCEM physical columns with valuation inputs derived from the Affective Valuation System (AVS), and we refer to this as the CEDeN. The circuit symbol of the CEDeN shown in Figure 3 and its fundamental operations of input integration, threshold determination, and output control were constructed on the basis of the neuronal computational circuits and circuit definitions presented in our previous studies (Tsubo, 2023, 2024).

**FIGURE 3:**
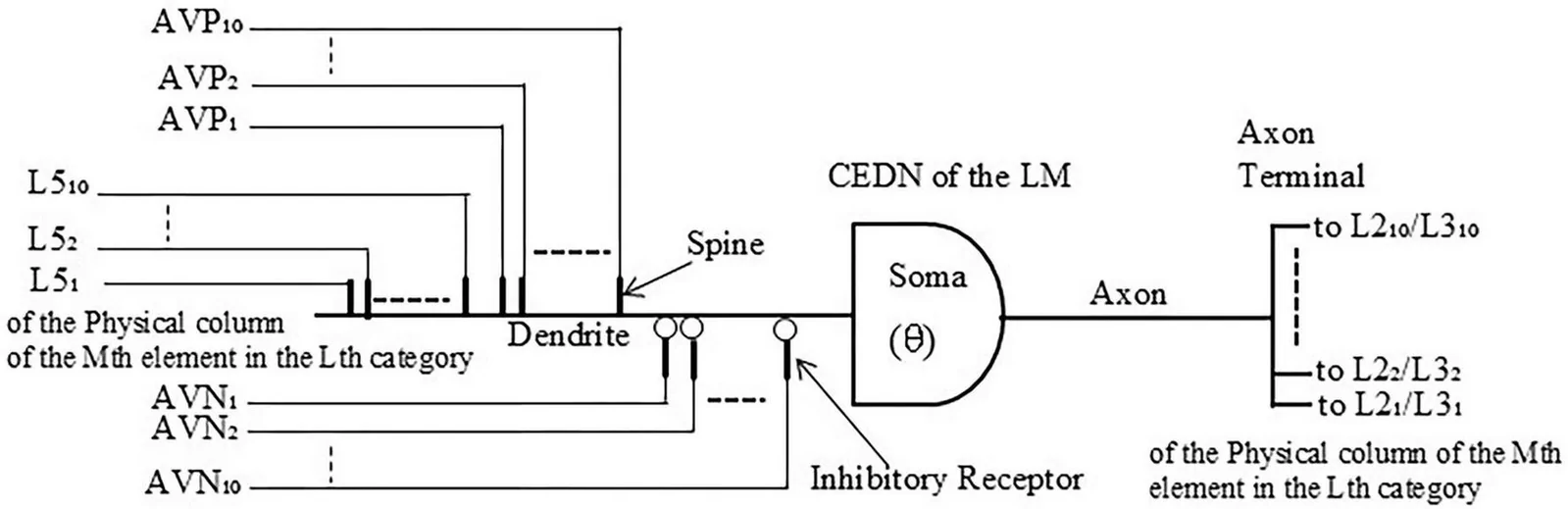
Conceptual circuit model of the CEDeN corresponding to the M-th contextual element (CE) in the L-th category. The CEDeN is assumed to exist within the thalamic MD nucleus. The long line extending leftward from the soma of the CEDeN represents a dendrite. Short line segments on the dendrite represent excitatory spine inputs, whereas short line segments with circles represent inhibitory receptor inputs. When the effective membrane potential, formed by the difference between the total amount of positive charge retained within the spines and the effective total amount of negative charge corresponding to inhibitory inputs, exceeds the functional threshold θ shown within the soma, the CEDeN generates an action potential and sends an axonal output. The output from the CEDeN is projected simultaneously to L2/3 of multiple DCEM physical columns constituting the corresponding logical column (of the CE). Through this simultaneously generated recurrent feedback, the L3 outputs of the multiple physical columns are delayed, as described in section “3.5 Local microcircuit dynamics underlying β-band periodicity.” The delayed L5 outputs resulting from these delayed L3 outputs then participate in the recurrent circuit, and the group of physical columns constituting the corresponding logical column (of the CE) forms and maintains a synchronized β-band CE Loop.

The total input I to the CEDeN corresponding to the Mth CE belonging to the Lth category is approximately expressed by the following equation.


I=(ΣL5+LMiΣAVP-LMiΣAVN)LMi×GNS≧LMθ

- ΣL5_LMi_: input from DCEM Layer 5 of the ith physical column constituting the LM CE (Douglas and Martin, 2004; Harris and Shepherd, 2015; Markram et al., 2015; Wang, 2013)
- ΣAVP_LMi_: the ith positive evaluation of the LM CE by the AVS (Aston-Jones and Cohen, 2005; Menon and Uddin, 2010; Padoa-Schioppa, 2011; Schultz, 2016)
- ΣAVN_LMi_: the ith negative evaluation of the LM CE by the AVS (Aston-Jones and Cohen, 2005; Menon and Uddin, 2010; Padoa-Schioppa, 2011; Schultz, 2016)
- GNS_LM_: global gain-modulatory effect of the GNS on the LM CE (Aston-Jones and Cohen, 2005; Avery and Krichmar, 2017; McCormick and Bal, 1997; Marder, 2012; Sara, 2009; Spitzer and Haegens, 2017)
- θ: threshold for CE Loop initiation

Here, ΣL5_LMi_ represents excitatory inputs from Layer 5 of the corresponding multiple DCEM physical columns, whereas ΣAVP_LMi_ and ΣAVN_LMi_ represent positive valuation input (AVP) and negative valuation input (AVN), respectively, from the AVS. GNS_LM_ represents the gain-modulatory effect of the Global Neuromodulatory System (GNS), and θ represents the functional firing threshold of the CEDeN. The biophysical framework by which a neuron integrates excitatory and inhibitory inputs and performs threshold determination was described in sections “3.2 Charge-retention properties of dendritic spines and extended synaptic integration” and “3.3 Computational functions of neurons” and in our previous studies (Tsubo, 2021, 2023, 2024). In contrast, the specific mechanism by which the CEDeN integrates these inputs at the level of individual CEs is a hypothesis newly proposed in the present study.

When the total input I exceeds the threshold θ, the CEDeN is assumed to fire and simultaneously send feedback to L2/3 of the multiple DCEM physical columns constituting the corresponding CE. This aligns the onset times of activity in the corresponding multiple physical columns, and the L3 firing delay produced by the L2/3 local circuit described in the next section, followed by the L5 firing delay, occurs synchronously across the multiple columns.

As a result, the relatively asynchronous γ-band candidate loops formed between DCEM and PAC/TAC are considered to transition to synchronized β-band CE Loops with longer periods, thereby maintaining the selected Contextual Element for a certain period.

The association of γ-band and β-band activities with different functional states is consistent with previous studies (Lundqvist et al., 2016, 2018; Spitzer and Haegens, 2017), whereas this CEDeN-dependent γ→β transition mechanism itself is a hypothesis specific to the DCEM model.

In contrast, when the content of PAC/TAC changes because of new sensory information, memory information, or behavioral outcomes and AVS evaluation shifts in a negative direction, resulting in increased AVN input, or when gain produced by the GNS decreases, the total input I to the CEDeN may fall below the threshold θ. As a result, the simultaneously generated feedback from the CEDeN ceases, and the corresponding β-band CE Loop is considered no longer maintainable.

In the present model, this threshold-mediated initiation, maintenance, and termination at the CE level are assumed to constitute one of the principal mechanisms enabling dynamic replacement of contextual content.

In this section, the CEDeN circuit is described as a static approximate model that does not explicitly include temporal dynamics, in order to clearly illustrate its logical structure. In practice, the spine inputs and inhibitory receptor inputs shown in Figure 3 are simplified functional representations of the retained-charge states and distributed input-integration properties described in sections “3.2 Charge-retention properties of dendritic spines and extended synaptic integration” and “3.3 Computational functions of neurons,” and the contribution of each input is considered to vary according to spine morphology, retained-charge state, and the spatiotemporal distributions of excitatory and inhibitory inputs (Tsubo, 2021, 2023, 2024).

In addition, although the present model assumes that the CEDeN performs threshold determination as a result of spatiotemporal integration of individual inputs, its detailed time-dependent dynamics and whether its actual neural implementation consists of a single neuron or a small functional neuronal population remain subjects for future experimental verification. The basic configuration concerning neuronal threshold computation and circuit symbols was presented in our previous studies (Tsubo, 2023, 2024).

### 3.5 Local microcircuit dynamics underlying β-band periodicity

In the present model, numerous transient candidate loops are considered to be formed through recurrent interactions between PAC/TAC and DCEM. Cortical γ-band activity is generally associated with rapid local and large-scale interactions and transient information representation (Buzsáki and Draguhn, 2004; Engel et al., 2001; Fries, 2005; Lundqvist et al., 2016, 2018; Singer, 1999), whereas β-band activity has been reported to be associated with the maintenance and reactivation of selected internal information (Lundqvist et al., 2016, 2018; Spitzer and Haegens, 2017). Against the background of these known frequency-band characteristics, DCEM assumes a transition from asynchronous γ-band candidate activity to β-band CE Loops synchronized at the CE level.

When synchronized feedback from the CEDeN is delivered to L2/3 of multiple DCEM physical columns constituting the same rogical column (same CE), these physical columns are considered to shift into a temporally aligned state. However, the synchronized input from the CEDeN itself is not assumed to directly impose the β-band periodicity from outside. In the present study, the relatively long period of the β-band is considered to be an intrinsic temporal structure formed by the L2/3 local circuitry and the biophysical properties of neurons within each physical column receiving synchronized input (Tsubo, 2021, 2023, 2024).

In the present model, when synchronized input is received from the CEDeN, L2 and L3 pyramidal neurons are assumed to begin depolarizing almost simultaneously. However, because L3 pyramidal neurons exceed L2 pyramidal neurons in dendritic structure, cell size, and number of synaptic inputs, the total membrane capacitance ΣCm of L3 pyramidal neurons is larger than that of L2 pyramidal neurons. Therefore, in response to simultaneous excitatory inputs of approximately the same magnitude, the increase in membrane potential in L3 is considered to occur more slowly than that in L2 (Tsubo, 2021, 2024).

As a result, L2 pyramidal neurons are considered to fire before L3 pyramidal neurons and to drive local inhibitory interneurons, particularly PV-positive interneurons that mediate rapid inhibition. It is widely supported that local inhibitory circuits including PV interneurons play important roles in the temporal structure of cortical activity and γ-band synchronization (Douglas and Martin, 2004; Fries, 2005; Harris and Shepherd, 2015; Singer, 1999).

In the present model, inhibitory input from PV interneurons driven by L2 to L3 is considered to transiently suppress the increase in L3 membrane potential. In the equivalent-charge model described in section “3.3 Computational functions of neurons,” this inhibitory effect is treated as an effective negative charge component corresponding to Cl^–^ influx and related effects (Tsubo, 2023, 2024). Meanwhile, because excitatory input from L2 to L3 continues in parallel, after the inhibitory effect decays, the excitatory electrical effect is considered to accumulate again and approach the firing condition (London and Häusser, 2005; Magee and Johnston, 2005; Stuart and Spruston, 2015; Tsubo, 2021).

When this reaccumulation causes the membrane potential of L3 to reach the functional threshold, L3 pyramidal neurons are considered to generate action potentials later than L2 pyramidal neurons.

Therefore, in the present model, the firing delay of L3 is considered to be reflected in a delay of L5 output. As a result, in multiple physical columns constituting the same CE, simultaneous feedback from the CEDeN initiates a common temporal sequence of L2 activity → local inhibition → L3 firing delay → L5 output delay. This delay prolongs the circulation time of the recurrent loops of the group of physical columns corresponding to that CE, while at the same time the CEDeN aligns the phases across multiple columns. Thus, the present study assumes a transition from relatively rapid and asynchronous γ-band candidate activity to synchronized β-band CE Loops with longer periods.

Accordingly, in the present model, β-band CE Loops are positioned not as structures formed solely by conduction delays between the cortex and thalamus, but as intrinsic periodic structures formed by the combination of simultaneous feedback from the CEDeN to multiple physical columns constituting the logical column of a CE and the local temporal dynamics of L2–inhibitory interneuron–L3–L5 within each physical column.

This specific local-circuit mechanism is currently a hypothesis and can be experimentally tested by simultaneous measurement of firing timing in L2/L3/L5, PV interneuron activity, and MD–cortical activity.

Taken together, the mechanisms of context formation, maintenance, and state transition in DCEM described in Chapters 1 and 2 may be explained stepwise from the neuronal level to the system level by combining the axonal conduction, spine charge retention, excitatory and inhibitory input integration, and temporal dynamics of local cortical circuits presented in this chapter (Tsubo and Kurokawa, 2018; Tsubo, 2021, 2023, 2024). In the next chapter, based on these biophysical foundations, we discuss the theoretical positioning of the DCEM model, its differences from existing theories, its experimental testability, and its limitations.

## 4 Discussion

The DCEM model proposed in this study is an integrative framework derived by coherently integrating established findings accumulated through EEG and MEG measurements of brain activity (Buzsáki and Draguhn, 2004; Engel et al., 2001; Fries, 2005), fMRI analyses of large-scale brain networks (Menon and Uddin, 2010; Miller and Cohen, 2001), neuroanatomical studies (Douglas and Martin, 2004; Griffiths et al., 2022; Harris and Shepherd, 2015), neurophysiological studies (London and Häusser, 2005; Singer, 1999; Stuart and Spruston, 2015), and studies of cortico-thalamic networks and working memory (Douglas et al., 1995; Halassa and Sherman, 2019; Lundqvist et al., 2016, 2018; Wang, 2001; Watanabe and Funahashi, 2012; Wolff and Halassa, 2024), with the authors’ previous biophysical studies of intrinsic neuronal properties (Tsubo and Kurokawa, 2018; Tsubo, 2021, 2023, 2024).

Chapters 1–3 presented the theoretical and biophysical basis underlying this integration. In this chapter, we discuss the theoretical position of DCEM emerging from this integration, its relationship to existing theories, its biophysical significance, its testability, and future challenges.

### 4.1 Positioning of the DCEM model and the aim of the present study

In this study, we proposed the Dynamic Contextual Element Memory (DCEM) model to explain the formation, maintenance, and updating of context in accordance with internal desires, as well as the generation of behavior based on that context, on the basis of intrinsic biophysical properties of neurons. As shown in the Introduction and Figure 1, the model assumes that continuously generated desires from the Affective Valuation System (AVS) provide the driving force for contextual formation, that contextual candidates are generated from the numerous inputs transmitted from PAC/TAC, and that, through selection and synchronization by CEDeNs, a contextual state that can be utilized for behavior is formed.

The DCEM model aims to explain the neural mechanisms by which contextual contents are formed, selected, maintained, updated, and terminated as mechanisms underlying transitions between conscious states. However, the present model does not explain why or how the contextual states formed through these mechanisms are subjectively experienced or become consciously recognized by the individual. The scope of the present study is therefore limited to the neural mechanisms underlying the formation and dynamic transitions of contextual contents and their transmission to language and behavioral output systems. Although the maintenance of information by CE Loops functionally overlaps in part with working memory, the two are not identical. Working memory temporarily maintains information that can be used for a variety of purposes, whereas each CE Loop in the DCEM framework corresponds to a specific CE and maintains the contextual content selected for that CE. Furthermore, individual CE Loops can be independently formed, maintained, terminated, and replaced according to AVS evaluation, thereby enabling continuous updating of the overall contextual state.

A distinctive feature of DCEM is that it does not define a conscious state as activity in a single brain region or within a particular frequency band itself. Rather, a conscious state is regarded as a dynamic contextual state formed through the selection, maintenance, termination, and replacement of multiple Contextual Elements (CEs) while being driven by the AVS. Thus, even while an overarching desire or goal persists, individual CEs required to achieve that desire or goal may be sequentially replaced in response to changes in sensory information, memory information, internal states, and behavioral outcomes. Through this continuous updating at the CE level, the overall context is considered to transition over time.

Furthermore, γ-band and β-band activities in the present model are not introduced as indicators of consciousness in themselves. As described in the Introduction, numerous asynchronous γ-band candidate loops correspond to a state in which information that may constitute a context is explored in parallel, whereas a β-band CE Loop, formed through synchronization of multiple physical columns constituting a selected CE, is positioned as a mechanism that ensures the temporal coherence of encoded contextual information and enables downstream behavior-related systems to decode that information appropriately.

Accordingly, the central proposal of the DCEM model is not to explain known neural phenomena such as γ-band activity, β-band activity, valuation, or neuromodulation individually. Rather, starting from the intrinsic biophysical properties of neurons and extending them to interactions among cortical columns, recurrent cortico-thalamic circuits, the AVS, and CEDeNs, the model integrates into a single neural mechanism the sequence of processes from internal desires to contextual formation, contextual-state transitions, and ultimately behavioral output.

Based on this theoretical positioning, the following sections discuss the biophysical basis of the DCEM model, its relationship to existing theories, the transition from γ-band candidate loops to β-band CE Loops, contextual formation driven by the AVS, the functional positioning of CEs, and the testable predictions derived from the model.

### 4.2 Intrinsic neuronal properties and the biophysical basis of the DCEM model

As described in Chapter 3, the DCEM model proposed in this study takes as its starting point the intrinsic biophysical properties of neurons that the authors have previously analyzed (Tsubo and Kurokawa, 2018; Tsubo, 2021, 2023, 2024). Because the sequence and progression of these studies and their biophysical basis were described in detail in Chapter 3, the present section focuses on their significance for the system-level mechanisms of DCEM.

These intrinsic neuronal properties are important for the DCEM model because they allow individual neurons to be treated not merely as instantaneous input–output elements, but as functional elements capable of temporally integrating numerous inputs and changing their outputs according to their integrated states (Tsubo and Kurokawa, 2018; Tsubo, 2021, 2023, 2024). In the DCEM model, such neuronal properties are repeatedly utilized within cortical columns and are further extended to cortico-cortical and cortico-thalamic circuits, thereby enabling the formation, selection, synchronization, maintenance, and updating of contextual candidates.

Accordingly, the biophysical characteristic of the DCEM model does not lie in simply combining individual components, such as γ-band activity, β-band activity, CEDeNs, or the AVS, as separate hypotheses. Rather, it lies in taking the neuronal-level properties described in Chapter 3 as the starting point, progressively extending them from local cortical circuits to cortico-thalamic networks, and linking them to a system-level mechanism that explains contextual formation and state transitions.

On the other hand, some of the biophysical mechanisms described in Chapter 3, particularly the mechanism of membrane-potential formation based on the relationship between intracellularly retained charge and total membrane capacitance, require further direct experimental verification. Thus, DCEM is a system-level model derived on the basis of these cellular-level hypotheses, while the underlying biophysical mechanisms themselves also have an experimentally testable structure.

### 4.3 Relationship to existing theories of consciousness and the originality of DCEM

The DCEM model is not intended to replace existing theories such as Global Workspace Theory (GWT), Integrated Information Theory (IIT), or Predictive Processing (PP; Dehaene and Changeux, 2011; Friston, 2010; Mashour et al., 2020; Tononi, 2008). Whereas these theories, respectively, emphasize such explanatory principles as global sharing of information associated with conscious access, information integration, or prediction and error correction, the DCEM model aims to explain the formation, selection, synchronization, maintenance, termination, and replacement of contextual candidates driven by internal desires as a neural mechanism extending from individual neurons to cortico-thalamic circuits, based on the intrinsic biophysical properties of neurons demonstrated in the authors’ previous studies (Tsubo and Kurokawa, 2018; Tsubo, 2021, 2023, 2024).

In GWT, ignition, in which selected information exceeds a threshold and becomes globally shared across widespread networks, is positioned as an important mechanism of conscious access (Dehaene and Changeux, 2011; Mashour et al., 2020). The CEDeN threshold mechanism in DCEM has a functional similarity to ignition in that activity exceeding a threshold enables subsequent large-scale neural activity. In the DCEM model, however, this threshold mechanism is assumed to operate separately for each logical CE. As shown in the Introduction and Figure 1, a CEDeN integrates L5 inputs from the corresponding group of physical columns with valuation inputs from the AVS and, when the threshold is exceeded, synchronizes the physical columns constituting that CE. Thus, the threshold mechanism in DCEM is not a single ignition mechanism for globally broadcasting an entire representation, but rather a selection and synchronization mechanism that forms a β-band CE Loop for each individual CE and controls its formation, maintenance, and termination.

IIT explains consciousness in relation to the intrinsic causal integration structure of a system (Tononi, 2008). In contrast, the DCEM model does not aim to quantify the amount or degree of integration of consciousness; rather, it addresses the temporal state-transition mechanism through which the CEs constituting a context at a given moment are selected and maintained, and subsequently terminated and replaced as conditions change. Thus, information integration in the DCEM model is not itself the primary explanatory target but is positioned as one component of the process that enables dynamic contextual formation.

In PP, perception and behavior are updated through hierarchical prediction, prediction error, and precision weighting (Friston, 2010). DCEM shares with PP the general property that states are updated on the basis of external information, internal information, and past experience. However, the DCEM model explicitly introduces desires and valuation continuously provided by the AVS as driving conditions for contextual formation. It further differs in proposing a specific circuit mechanism through which individual CEs are selected on the basis of such valuation, formed and maintained as β-band CE Loops, and terminated and replaced when the valuation changes.

There are also functional similarities between DCEM and working-memory or attractor-network models (Amit, 1995; Baddeley, 2003; Goldman-Rakic, 1995; Hopfield, 1982; Lundqvist et al., 2016, 2018). In particular, the maintenance of information selected by β-band CE Loops for a certain period resembles persistent activity or stable network states. In the DCEM model, however, information maintenance itself is not the ultimate objective.

As described in the Introduction, the model assumes that temporal coherence among multiple encoded pieces of information is necessary for downstream systems to decode them appropriately, and β-band CE Loops are positioned as a mechanism for establishing this synchronized state. Furthermore, because individual CE Loops can be independently formed, maintained, terminated, and replaced, the specific contextual contents required to achieve an overarching goal or desire can be continuously updated even while that goal or desire generated by the AVS persists.

Thus, the originality of the DCEM model does not reside in individual phenomena such as γ-band activity, β-band activity, valuation, neuromodulation, or cortico-thalamic interactions themselves. Rather, based on the intrinsic biophysical properties of neurons described in Chapter 3, its originality lies in linking the formation of numerous asynchronous γ-band candidate loops, valuation by the AVS, CE-level threshold selection by CEDeNs, and synchronization, maintenance, termination, and replacement through β-band CE Loops into a single neural mechanism that explains the process from internal desires to dynamic contextual formation and behavioral output. Experimental predictions and falsification criteria derived from this specific mechanism are described in section “4.8 Testable predictions and falsifiability.”

### 4.4 Functional transition from γ-band candidate loops to β-band CE loops

In this study, γ-band and β-band activities are positioned not as mutually independent neural phenomena, but as different stages of a continuous neural process extending from the exploration of contextual candidates to the synchronization, maintenance, and behavioral utilization of selected contextual information (Buzsáki and Draguhn, 2004; Fries, 2005; Lundqvist et al., 2016, 2018; Spitzer and Haegens, 2017). The specific circuit organization and formation process were described in the Introduction and Figure 1.

The functional significance of numerous asynchronous γ-band candidate loops is that diverse information transmitted from PAC/TAC can be maintained and explored in parallel as multiple candidates for individual CEs without immediately fixing that information into a single context. However, while these candidates remain mutually asynchronous, it would be difficult for downstream systems to stably decode information distributed and encoded across multiple physical columns while preserving temporal coherence. The DCEM model therefore assumes that, following candidate formation, a second stage is required in which the necessary information is selected and synchronized.

This role is assigned to selection and synchronous feedback by CEDeNs and to the β-band CE Loops formed as a result. As described in the Introduction and section “3.5 Local microcircuit dynamics underlying β-band periodicity,” when the integrated input to a CEDeN, including valuation by the AVS, exceeds its functional threshold, the CEDeN synchronizes the multiple physical columns constituting the corresponding logical CE. Consequently, a subset of the information that had previously existed as asynchronous candidates is considered to transition into a temporally coherent β-band CE Loop.

The important significance of this γ-to-β transition is not simply a reduction in oscillation frequency, but a functional transformation from a candidate state into a selected, synchronized, and usable contextual state. DCEM Layer 5 outputs synchronized through β-band CE Loops are considered to ensure the temporal coherence of distributed encoded contextual information, thereby enabling downstream systems involved in behavior, language generation, and motor control to decode that information appropriately.

Furthermore, β-band CE Loops are not mechanisms for permanently maintaining a fixed context. As described in the Introduction, individual CE Loops can be independently formed, maintained, terminated, and replaced. Thus, even while an overarching goal or desire generated by the AVS persists, the specific CE contents required to achieve that goal can be sequentially updated in response to changes in sensory information, memory information, internal states, and behavioral outcomes.

This property allows the maintenance of a stable goal to coexist with flexible contextual contents that change from moment to moment.

Thus, the γ→β transition in the DCEM model is positioned as a functional transition linking exploration → selection → synchronization → utilization → updating. By repeatedly executing this sequence at the CE level, the overall context continuously changes and corresponding behavior is generated. This constitutes the principal functional significance of γ-band candidate loops and β-band CE Loops in the present model.

### 4.5 AVS-driven contextual-state transitions and adaptive value formation

In the present model, the Affective Valuation System (AVS) is positioned not simply as a system that retrospectively evaluates an already formed context, but as a functional organization that provides desire- and value-related signals based on biological needs and experience-dependent values and thereby drives contextual formation by DCEM (Aston-Jones and Cohen, 2005; Menon and Uddin, 2010; Padoa-Schioppa, 2011; Schultz, 2016).

As shown in the Introduction and Figure 1, valuation by the AVS participates in CE selection through CEDeNs, and changes in the valuation state of the AVS are considered to alter the combination of CEs that are formed and maintained.

An important feature of this organization is the distinction between persistence of an overarching desire or goal generated by the AVS and persistence of the specific contextual contents required to achieve that desire. For example, even when an AVS driving state corresponding to a particular goal persists for a relatively long period, sensory information, memory information, internal states, and behavioral outcomes change over time. Therefore, even while the same goal is maintained, β-band CE Loops corresponding to individual CEs can be independently terminated and replaced, allowing the specific contextual contents to be continuously updated. This mechanism is considered to reconcile persistence of a goal with flexible updating of context in response to environmental changes.

Furthermore, the role of the AVS is not limited to evaluating current contextual candidates. When behavior is executed on the basis of a selected context, the resulting changes in the external environment and internal state subsequently influence valuation (Padoa-Schioppa, 2011; Schultz, 2016). Thus, the AVS may function as part of a feedback mechanism through which the extent to which a behavioral outcome satisfies the current desire influences subsequent valuation and contextual selection.

Such experience-dependent updating of value is also important for understanding the relationship between the innate Proto-AVS and higher-order AVS in the present model. While basic values directly related to survival provide the foundation, new valuations may be formed and modified through postnatal experience and learning, allowing different contexts to be selected in response to the same external information depending on an individual’s experience (Eichenbaum, 2017; Feldman, 2012; Kasai et al., 2010; Padoa-Schioppa, 2011; Schultz, 2016).

However, the present study does not aim to explain the process by which specific developmental experiences give rise to specific psychological characteristics; rather, the discussion is limited to the functional relationship whereby experience-dependent changes in value can influence contextual formation by DCEM.

Thus, the AVS in DCEM is considered to include the functions of driving contextual formation through internal desires, evaluating current candidate information according to value, and adaptively incorporating behavioral outcomes into subsequent valuation. These are not independent processes but constitute a cyclic interaction of desire → contextual formation → behavior → outcome → updating of valuation → subsequent contextual formation. Through this cyclic organization, DCEM can continuously update contextual states while reflecting both persistent desires and information that changes from moment to moment, rather than repeatedly reproducing a fixed context.

### 4.6 Definition of contextual elements and specification of their contents

In the DCEM model, Subject, Verb, Object, Complement, When, Where, Why, and How (Attribute) are assumed as representative Contextual Element Categories (CE Categories) that constitute context. However, these are not intended to map natural-language grammatical categories themselves onto the nervous system, but are hypothetical categories introduced to organize the functional roles that constitute the context required to translate AVS desires into action. Therefore, the present model does not assume that the conscious state itself is decomposed into a linguistic structure.

It is also necessary to distinguish among a CE Category, an individual Contextual Element (CE), and the specific information content corresponding to a CE. As shown in the Introduction and Table 1, each CE Category is assumed to contain multiple CEs, and each CE may correspond to specific information concerning perception, objects, actions, time, place, reasons, attributes, and related contents distributed across PAC/TAC.

The configuration assumed in the present model–approximately 200 logical CEs, approximately 25 CEs per category, and approximately 10 physical columns per logical CE–is a model assumption introduced to concretize this functional relationship and is not an established neuroanatomical constant (Mountcastle, 1997; Petersen, 2007).

The specific content of each CE is not fixed but is dynamically determined according to sensory information, memory information, internal information, and desires and evaluations from the AVS at that time. PAC/TAC contains a large amount of distributed information related to past knowledge, experience, sensory representations, and current external inputs (Eichenbaum, 2017; Fusi et al., 2016; Hickok and Poeppel, 2003; Rigotti et al., 2013), and information relevant to the current context is transmitted from among these as candidates to DCEM.

In the present model, through the formation of γ-band candidate loops and selection by CEDeNs described in the Introduction, part of this information is considered to be selected as the specific content of a particular CE.

This organization makes it possible to form diverse contexts from a limited number of CEs. Even for the same CE, the corresponding information content in PAC/TAC may differ depending on the situation. In addition, changes in the combinations of contents across multiple CEs alter the overall context that is formed. Therefore, a CE in DCEM should not be understood as representing a specific word, object, or fixed informational content, but rather as a functional structural unit that positions information within a dynamic context.

This point is also important in considering the relationship between the DCEM model and language. In the present model, language is not considered a prerequisite for context formation itself. Nonlinguistic perception, action, spatial information, temporal information, and related information can also constitute the specific content of CEs. Language generation is positioned as a process in which a contextual structure already formed within DCEM is converted by the language system into a temporal sequence for external expression (Fedorenko and Blank, 2020; Hickok and Poeppel, 2003). Thus, although CE Categories may partially correspond to linguistic structures, context formation by DCEM itself does not presuppose the existence of language ability.

Taken together, CEs are not proposed as information-representation units intended to replace large-scale nonlinear neural networks, but as structural units for functionally describing contexts formed through dynamic interactions among PAC/TAC, DCEM, and cortico-thalamic systems. By distinguishing CE Categories, CEs, and the specific informational content corresponding to individual CEs, the DCEM model can describe the process by which dynamic contexts are formed from diverse sensory, memory, and internal information without reducing consciousness to fixed linguistic categories.

### 4.7 Overall significance of the DCEM framework

The principal significance of the DCEM model proposed in the present study lies in presenting a framework that begins with the intrinsic biophysical properties of neurons and links local cortical circuits, cortico-thalamic interactions, context formation, conscious-state transitions, and behavioral output as a continuous neural process (Tsubo and Kurokawa, 2018; Tsubo, 2021, 2023, 2024). Whereas existing theories of consciousness have mainly explained information sharing, information integration, or predictive processing at the network level (Dehaene and Changeux, 2011; Friston, 2010; Mashour et al., 2020; Tononi, 2008), the DCEM model approaches the problem from a different direction, aiming to bridge the intrinsic properties of neurons and dynamic neural activity at the system level.

Within this framework, context formation is not established by a single brain region or a single type of neural activity, but through interactions among PAC/TAC, DCEM, the AVS, and CEDeNs hypothesized to exist within the thalamic MD nucleus. As shown in the Introduction and the preceding sections, necessary CEs are selected from numerous asynchronous γ-band candidate loops, and the corresponding groups of physical columns are formed as synchronized β-band CE Loops. Multiple CE Loops are then maintained in parallel to form the context at a given moment, and contextual states are considered to transition continuously through the termination and replacement of individual CE Loops. This organization makes it possible to treat internal desires, information exploration, selection, synchronization, context formation, and behavioral output as a series of dynamic neural processes.

Another characteristic of the DCEM model is that it can explain stable goals and flexible contextual updating within the same framework. Even when a major desire or goal reflected in the AVS persists, individual CE Loops can be sequentially terminated and replaced in response to changes in sensory information, memory information, internal states, and behavioral outcomes.

Thus, an organism can maintain a single goal while continuously changing the specific contextual content required to achieve that goal. In DCEM, this dynamic updating process is positioned as an important neural mechanism constituting conscious-state transitions.

On the other hand, the present model includes hypotheses that require future experimental verification, including the existence and function of CEDeNs, the correspondence between logical CEs and physical columns, valuation inputs from the AVS to CEDeNs, and the transition mechanism from γ-band candidate loops to β-band CE Loops. Although previous studies have shown that the MD nucleus plays important roles in recurrent interactions with the prefrontal cortex and in cognitive control (Griffiths et al., 2022; Halassa and Kastner, 2017; Halassa and Sherman, 2019; Sherman, 2016; Watanabe and Funahashi, 2012; Wolff and Halassa, 2024), the existence of CEDeNs operating for individual CEs and their specific synchronized-feedback mechanism are anatomical and functional hypotheses proposed in the present study.

Accordingly, the DCEM model is positioned not as a completed neurophysiological explanation, but as a testable biophysical framework linking the neuronal level to the system level. Its specific experimental predictions and falsification criteria are described in section “4.8 Testable predictions and falsifiability.”

Furthermore, although the central subjects of the present study are context formation and conscious-state transitions, this framework may provide a common basis for considering their functional relationships with memory, language, and learning. Specifically, the reflection of formed contexts into memory, linguistic output of already formed contextual structures, and changes in valuation and context formation accompanying experience can be examined as functions derived from the fundamental context-formation mechanism of DCEM. However, whether these higher-order cognitive functions can be comprehensively explained by the DCEM model requires independent experimental and theoretical examination in future studies.

Taken together, the DCEM model is considered to provide a testable theoretical framework that links the intrinsic biophysical properties of neurons to large-scale cortico-thalamic network activity and explains dynamic context formation and conscious-state transitions based on internal desires.

### 4.8 Testable predictions and falsifiability

The DCEM model proposed in the present study includes hypotheses that currently require experimental verification, including the existence of CEDeNs, the transition from γ-band candidate loops to β-band CE Loops, CE selection by the AVS, and contextual updating at the CE level. Because these are major components required for the model to function, experimental predictions can be established for each, and findings inconsistent with these predictions would require modification of the corresponding hypothesis or of the DCEM model itself.

First, DCEM predicts that during the exploration stage of contextual candidates, numerous candidate loops including the recurrent pathway


PAC/TACL⁢2/3→DCEML2/3→DCEML5

→PAC/TACL2/3

are formed and exist in parallel as γ-band activities that are not synchronized with one another. The association of γ-band activity with transient information representation and candidate activity is consistent with previous studies (Fries, 2005; Lundqvist et al., 2016, 2018; Spitzer and Haegens, 2017). Furthermore, when new sensory information, memory information, or internal information is added, new candidate loops are predicted to form while some previously active candidate loops terminate, thereby dynamically updating the candidate set. If such dynamic formation and termination of candidate loops are not observed and contextual selection is shown to be established through a different circuit mechanism, the γ-band candidate-formation mechanism of the DCEM model would require modification.

Second, the DCEM model predicts that the mediodorsal (MD) thalamic nucleus contains Contextual Element Decision Neurons (CEDeNs), or functionally equivalent neuronal populations, corresponding to individual logical CEs (Griffiths et al., 2022; Halassa and Kastner, 2017; Halassa and Sherman, 2019; Sherman, 2016; Watanabe and Funahashi, 2012; Wolff and Halassa, 2024). Each CEDeN is hypothesized to integrate Layer 5 inputs from the multiple physical columns constituting the corresponding logical CE with positive and negative valuation inputs from the AVS. A critical causal prediction of the model is that, if cortical gamma-band candidate activity corresponding to a particular CE is preserved while the activity of its corresponding CEDeN is selectively suppressed, the gamma candidate activity should remain, whereas its transition to synchronized beta-band activity and the formation of the corresponding CE Loop should be selectively impaired. Conversely, selective activation of the corresponding CEDeN, when the relevant gamma-band candidate activity is present, should facilitate the gamma-to-beta transition and formation of the corresponding CE Loop. Furthermore, when several gamma-band candidate activities coexist, selective manipulation of the CEDeN corresponding to one candidate should selectively affect the gamma-to-beta transition of that candidate without necessarily disrupting the other candidates. Thus, the causal dissociation between preserved cortical gamma-band candidate activity and the selective loss or facilitation of the corresponding beta-band CE Loop provides a direct and distinctive test of the proposed role of CEDeN in the DCEM model. If selective manipulation of MD/CEDeN activity does not selectively affect the gamma-to-beta transition and formation of the corresponding CE Loop, the proposed CEDeN-dependent selection mechanism would require revision.

Third, the DCEM model predicts that synchronized input from a CEDeN synchronizes the activity of multiple physical columns constituting the same logical CE and causes a transition from a γ-band candidate state to a β-band CE Loop. This transition is considered to involve a delay in L3 output generated by the L2–PV–L3–L5 local circuit described in section “3.5 Local microcircuit dynamics underlying β-band periodicity,” resulting in a longer period of synchronized L5 output than in the γ-band. This local-circuit mechanism is a hypothesis derived in the present study on the basis of the neuronal biophysical properties and computational mechanisms analyzed in the authors’ previous studies (Tsubo, 2021, 2023, 2024).

Accordingly, if the predicted synchronized feedback accompanying CE selection occurs without corresponding delays in L3 and L5 activity, or if β-band CE Loops are shown to form independently of this mechanism, the γ→β transition mechanism of the present model would require modification.

Fourth, the present model predicts that after a β-band CE Loop is formed, L5 outputs from multiple physical columns constituting the same logical CE become synchronized, allowing these inputs to be temporally summed in the CEDeN and reducing the dependence of Loop maintenance on AVP input from the AVS. In contrast, when AVN input from the AVS increases and the integrated value of the CEDeN falls below the functional threshold, synchronized output from the CEDeN is predicted to stop, and the corresponding β-band CE Loop is also predicted to terminate.

Therefore, if maintenance of β-band CE Loops is shown to require continuous AVP input, or if an increase in AVN does not selectively terminate the corresponding CE Loop, the maintenance and termination mechanism of CE Loops would require reconsideration.

Fifth, the DCEM model predicts that β-band CE Loops corresponding to multiple CEs are maintained simultaneously and that their combination forms the contextual state at a given moment. Because individual CE Loops can be independently formed, maintained, terminated, and replaced, the specific CE contents required to achieve a higher-order desire or goal are predicted to change continuously even while AVS activity corresponding to that desire or goal persists. Thus, maintenance of the goal together with continuous CE-level updating of the contextual content required to achieve it is a prediction characteristic of the present model. If such selective updating at the CE level is not observed and the entire context is shown to be formed, maintained, and replaced only as an integrated whole, the CE-level context-composition hypothesis of the present model would require reconsideration.

Sixth, the present model assumes that L5 outputs from multiple physical columns synchronized through β-band CE Loops transmit coded contextual information to downstream output systems while maintaining temporal coherence. Previous studies have reported that β-band activity is related to the maintenance and reactivation of selected internal information (Lundqvist et al., 2016, 2018; Spitzer and Haegens, 2017), whereas the DCEM model positions this synchronization as a specific mechanism for ensuring the temporal coherence of distributed contextual information.

Therefore, selective disruption of β-band synchronization corresponding to a particular CE is predicted to selectively impair decoding of information related to that CE in downstream systems or the corresponding behavioral output. In contrast, if decoding of the corresponding information and behavioral output remain normal despite disruption of β-band synchronization, the hypothesis that positions β-band CE Loops as a synchronized decoding mechanism would require reconsideration.

These predictions may be experimentally tested in the future using laminar multisite electrophysiological recordings, simultaneous measurements of the thalamic MD nucleus and cortex, cell-type-selective manipulations, and large-scale neural-activity measurements with high temporal resolution. In particular, if γ-band candidate activity between PAC/TAC and DCEM, MD-nucleus activity corresponding to CEDeNs, synchronization of cortical column groups following CEDeN output, delays in L3–L5 activity, formation, maintenance, and termination of β-band CE Loops, and replacement of contextual content at the CE level can be tracked temporally within the same task, the principal causal relationships of the DCEM model could be directly tested.

Thus, the DCEM model is not merely a model that retrospectively explains observed γ-band or β-band activity; rather, it presents specific predictions concerning the circuits in which these activities are formed, how they are selected and synchronized, under what conditions they are maintained, and under what conditions they terminate. Therefore, experimental findings inconsistent with these predictions would require modification of the corresponding component hypotheses, and if multiple central predictions were rejected, the DCEM framework itself would require modification or reconsideration.

## 5 Conclusion

In the present study, we organized the intrinsic biophysical properties of neurons analyzed in our series of previous studies (Tsubo and Kurokawa, 2018; Tsubo, 2021, 2023, 2024) and clarified them in section “1.1 Fundamental properties of neurons” as Fundamental Properties 1–5 of neurons that are important for understanding the formation, roles, and operation of neuronal networks. Furthermore, using these fundamental properties as a common axis, we functionally related the extensive neuroscientific findings that have accumulated across different hierarchical levels and research fields, including neurons, interlaminar cortical circuits, cortical columns, cortico-thalamic interactions, neural oscillations, valuation, and neuromodulation.

As a result, we derived the Dynamic Contextual Element Memory (DCEM) model as a biophysical framework extending continuously from the neuronal level through local cortical circuits and cortico-thalamic networks to context formation, conscious-state transitions, and behavioral output.

From this integration, the present study proposed multiple interrelated theoretical constructs within the DCEM framework, while functionally organizing established valuation-related neural circuitry as the Affective Valuation System (AVS). In this framework, the AVS provides desire- and value-related signals based on biological needs and experience-dependent values and thereby drives contextual formation. The newly proposed theoretical constructs include DCEM, which constructs context as combinations of multiple Contextual Elements (CEs); exploration of candidate CE contents through numerous asynchronous γ-band recurrent activities; CE-level selection by the Contextual Element Decision Neuron (CEDeN), hypothesized to exist within the thalamic MD nucleus; and β-band CE Loops formed through synchronization of selected groups of physical columns. Furthermore, β-band CE Loops are positioned as synchronization mechanisms that maintain selected CEs while transmitting coded contextual information to downstream output systems in a temporally coherent state.

Through this organization, the context at a given moment is formed as a combination of β-band CE Loops corresponding to multiple CEs, and individual CE Loops can be independently formed, maintained, terminated, and replaced. Therefore, even when a major desire or goal from the AVS persists, the specific CE contents required to achieve that goal can be sequentially updated according to changes in sensory information, memory information, internal states, and behavioral outcomes.

In the present model, this coexistence of “persistence of a goal” and “continuous updating of specific contextual contents” is positioned as an important characteristic enabling dynamic context formation and conscious-state transitions.

On the other hand, the present model includes hypotheses requiring future experimental verification, including the existence and function of CEDeNs, the correspondence between logical CEs and physical columns, the relationship between AVS valuation and CE selection, and the mechanism of transition from γ-band candidate activity to β-band CE Loops. These represent limitations of the DCEM model, while at the same time providing specific targets for experimentally testing or falsifying the model.

Taken together, the novelty of the present study lies not in the discovery of individual neural phenomena themselves, but in identifying functional relationships among the extensive neuroscientific findings accumulated over many years across different research fields and hierarchical levels, using fundamental neuronal properties as a common axis, and integrating them into a continuous neural process extending from internal desires through candidate exploration, value-based selection, synchronized context formation, contextual-state transitions, and behavioral output. If the principal predictions presented in the present study are supported by future experimental research, the DCEM model may provide a new biophysical theoretical foundation linking fundamental neuronal-level properties with system-level dynamic context formation and conscious-state transitions.

## Data Availability

This study is a theoretical work and does not include newly generated or analyzed datasets. All information supporting the conclusions of this article is included in the article and its cited references. Further inquiries can be directed to the corresponding author.
